# Supplementary material for: In toto analysis of embryonic organisation reduces tissue diversity to two archetypes requiring specific cadherins
Source: Nat Commun. 2025 Jul 25;16:6872. doi: 10.1038/s41467-025-62127-9 (PMC12297461; doi:10.1038/s41467-025-62127-9)
Supplement: Supplementary file 1 — Supplementary Information [file 41467_2025_62127_MOESM1_ESM.pdf]

Supplementary Information

In toto multicellular organisation analysis reduces embryonic tissue diversity to two archetypes requiring cadherin expression

Max Brambach*, Jana Wittmann, Marvin Albert, Jérôme Julmi, Robert Bill, Darren Gilmour*

Department of Molecular Life Sciences, University of Zurich, Switzerland

* Correspondence: max.brambach@uzh.ch, darren.gilmour@uzh.ch

Supplementary Figures

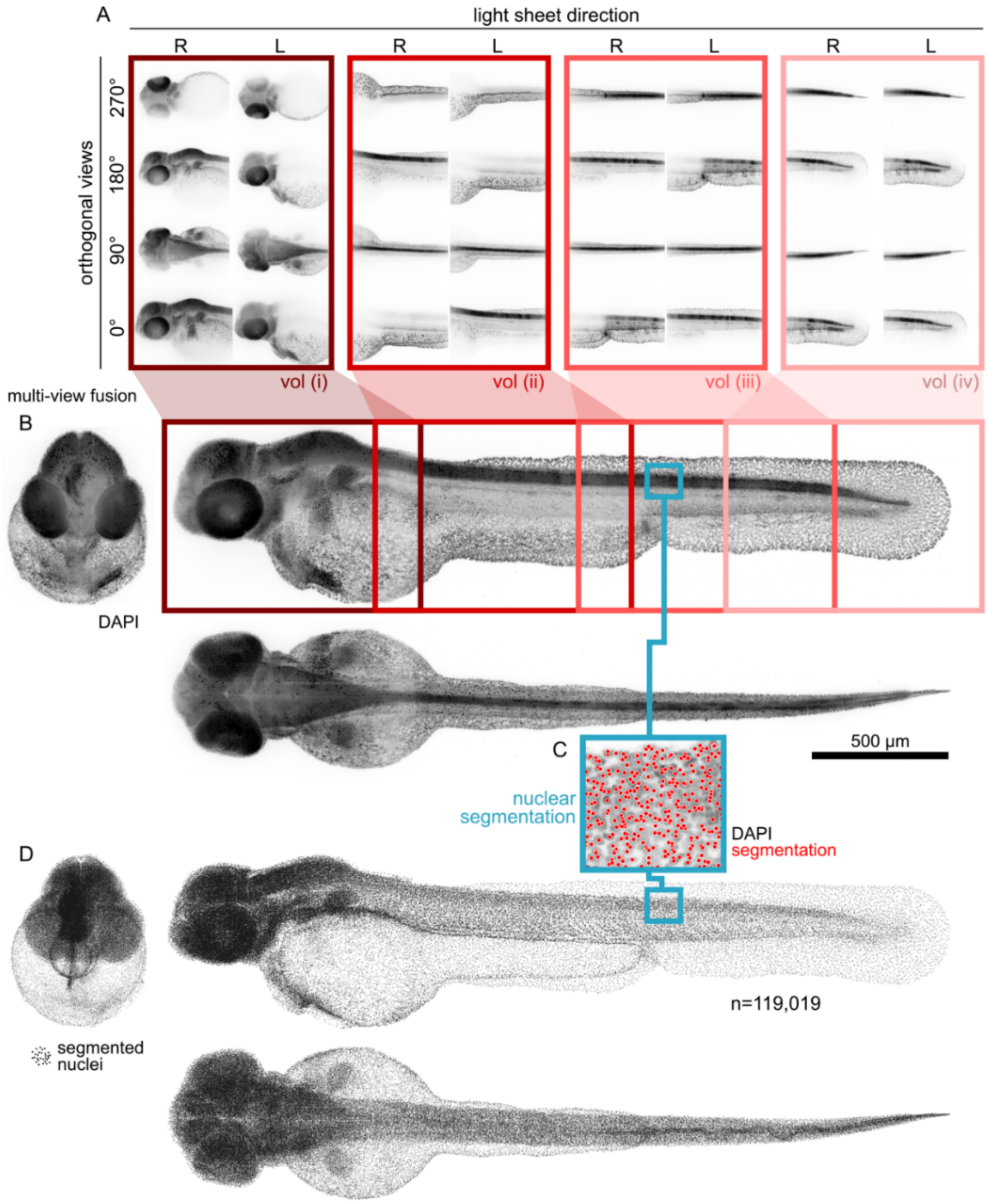

Supplementary Figure 1: Illustration of multi-view light sheet microscopy and nuclear segmentation pipeline

A: Raw images of zebrafish embryos at 48 hpf with DAPI-labelled nuclei were acquired using multi-view light sheet microscopy. 32 different volumetric image stacks were acquired per sample. Those consisted of four different, overlapping imaging volumes (vol (i) – vol (iv)) with four orthogonal views each (0° – 270°) and two opposing light sheet directions (R, L). **B**: Stacks were iteratively fused using Contrast Limited Adaptive Histogram Equalization[1] and Lucy-Richardson deconvolution[2,3], generating one volumetric image of the whole embryo consisting of the highest contrast regions of individual stacks (Albert, 2021). **C**: Individual nuclei were localised across the whole fused image using an iterative, multi-level watershed transformation and Gaussian Mixture Models[4,5]. Shallow maximum intensity projection across 50 µm. **D**: Point cloud representation of the sample. Locations of the segmented nuclei rendered as points. Images in A, B, D are whole-stack maximum intensity projections.

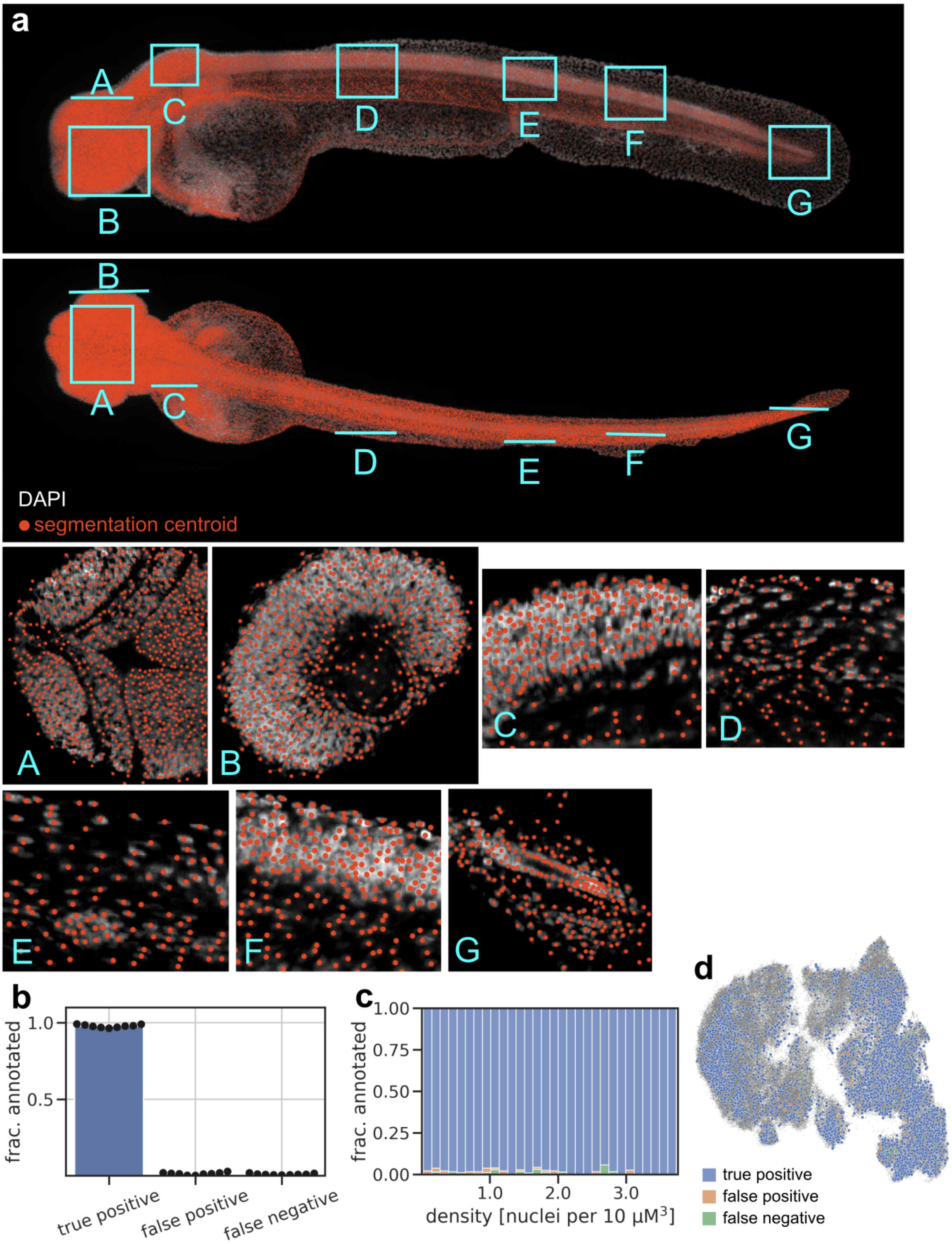
a
A
B
C
D
E
F
G
DAPI
segmentation centroid
b
frac. annotated
1.0
0.5
true positive
false positive
false negative
c
frac. annotated
1.00
0.75
0.50
0.25
0.00
1.0
2.0
3.0
density [nuclei per 10 μM^3]
d
true positive
false positive
false negative

Supplementary Figure 2: Nuclear segmentation is robust and accurate

a: Maximum intensity projections of 48 hpf zebrafish embryo labelled with DAPI overlayed with centroids of the nuclear segmentation. Panels A-G show single slice magnifications of individual areas to illustrate segmentation accuracy and robustness. **b**: Quantification of segmentation quality. Random fields of views of several samples were annotated manually to assess the rates of true positive, false positive and false negative identified nuclei. True positive rate: 97.8 ± 0.9 %; false positive rate: 1.3 ± 0.8 %; false negative rate: 0.9 ± 0.5 %. Dots represent individual fields of view. **c**: Segmentation quality does not depend on density. Shown as density is the multi-scale density (radius 30 μm) feature. **d**: Segmentation errors do not exhibit bias towards specific organisational patterns. t-SNE representation of the organisational feature space with manually annotated nuclei for segmentation validation overlayed. Number of manually annotated nuclei n=4212, from 9 randomly selected fields of view across N=3 randomly selected samples. Source data are provided as a Source Data file.

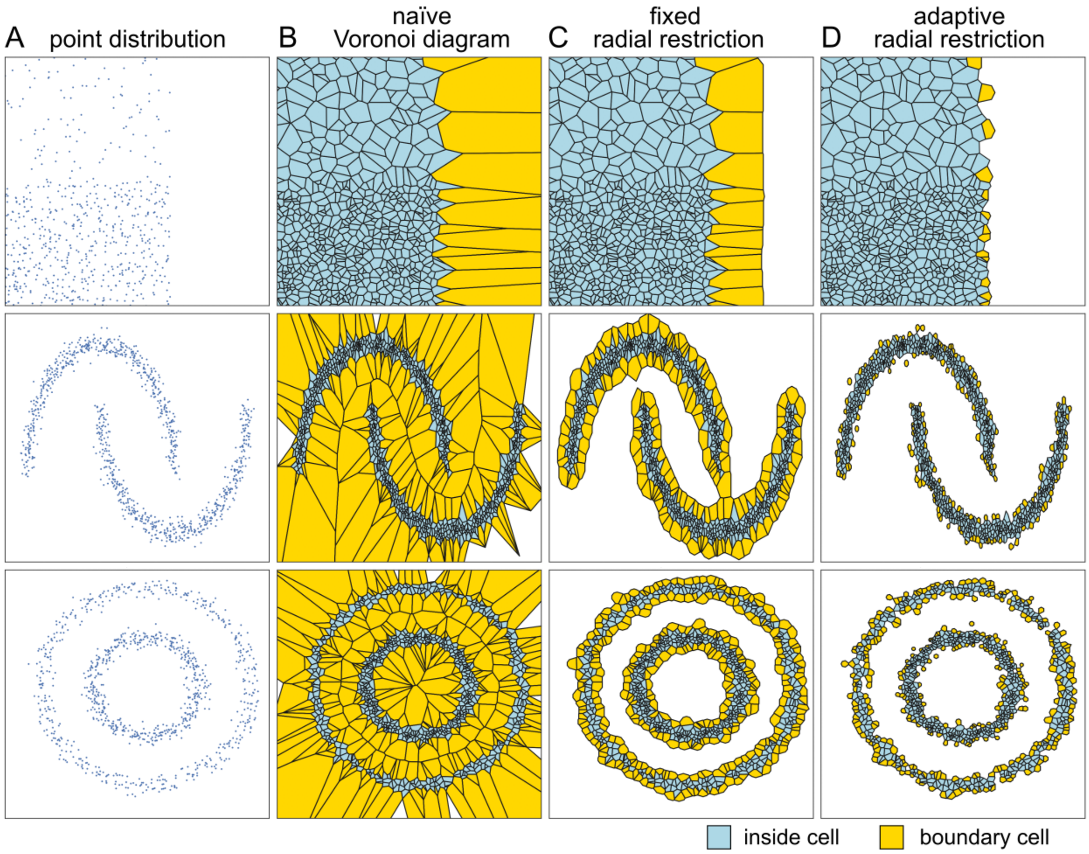

Supplementary Figure 3: Illustration of adaptively restricted Voronoi diagram

A: Distribution of points that generate the Voronoi diagrams. **B**: Naïve, unrestricted Voronoi diagram. **C**: Voronoi diagram with boundary cells restricted by a maximum radius. **D**: Adaptively restricted Voronoi diagram. In all datasets the naïve Voronoi diagram generates infinitely large boundary cells on the outside and connects unconnected regions on the inside. **Top**: Uniformly random distributed points of low (top) and high (bottom) density. Fixed radially restricted boundary cells are too large, and extend homogeneously to the outside, creating the impression of a smooth surface. Adaptively restricted boundary cells are larger in sparse and smaller in dense regions continuing the shape and size of their neighbouring cells. **Middle**: Double-crescent shaped random point distributions. Radially restricted boundary cells still connect between the crescents and are biased to similar size. Adaptively restricted boundary cells do not connect the crescents and generate isolated cells for points that are too distant from the main objects. **Bottom**: Concentric circle shaped random point distributions. Radially restricted boundary cells are of similar size, while adaptively restricted boundary cells are smaller in the denser inner ring. Note that the shown illustrations were calculated in 2D, while all analysis in this work was performed in 3D.

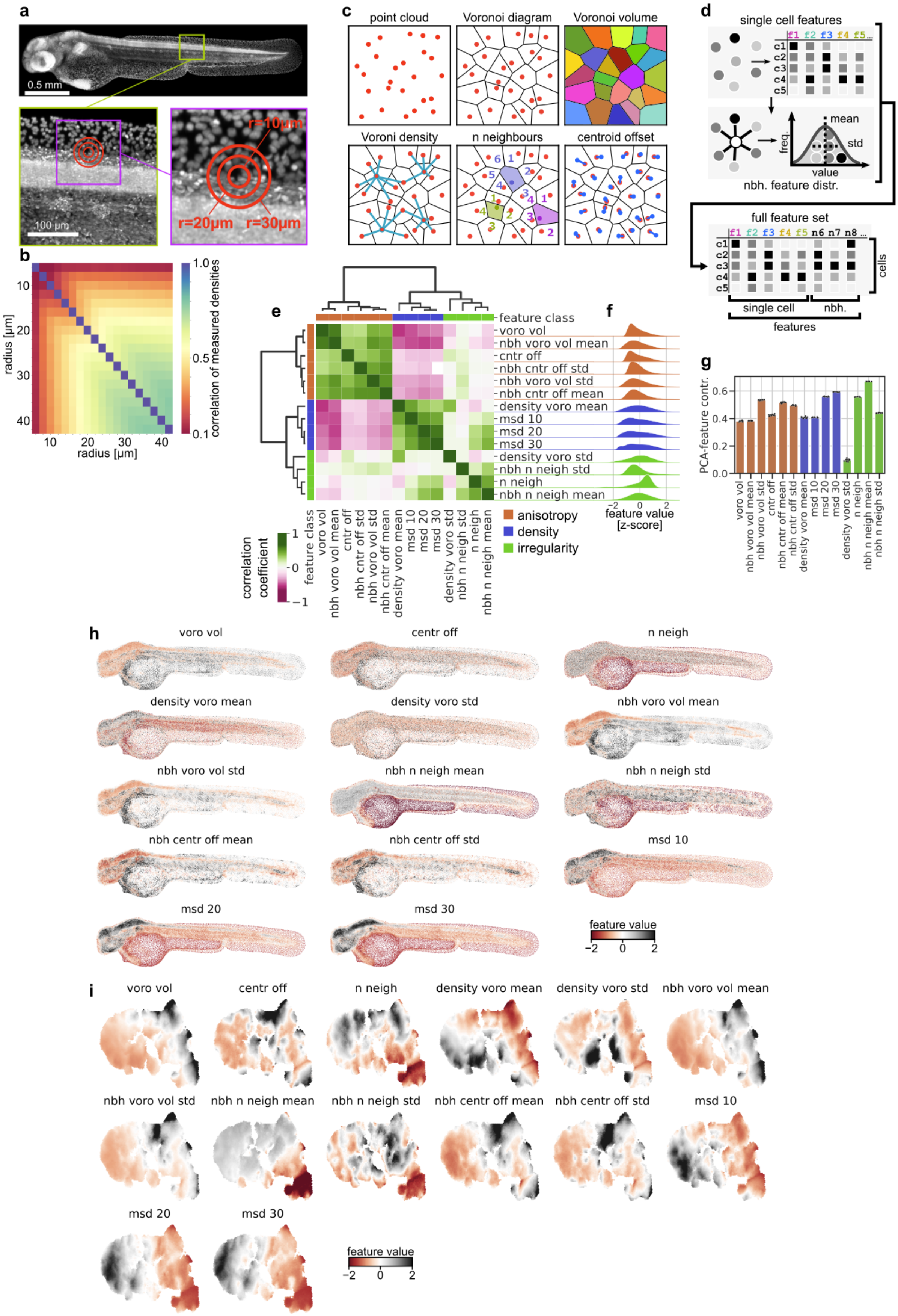

a
0.5 mm
100 µm
r=10µm
r=20µm
r=30µm
b
radius [µm]
correlation of measured densities
c
point cloud
Voronoi diagram
Voronoi volume
Voronoi density
n neighbours
centroid offset
d
single cell features
nbh. feature distr.
mean
std
freq.
value
full feature set
single cell
nbh.
features
cells
e
feature class
voro vol
nbh voro vol mean
cntr off
nbh cntr off std
nbh voro vol std
nbh cntr off mean
density voro mean
msd 10
msd 20
msd 30
density voro std
nbh n neigh std
n neigh
nbh n neigh mean
correlation coefficient
f
feature value [z-score]
anisotropy
density
irregularity
g
PCA-feature contr.
h
voro vol
centr off
n neigh
density voro mean
density voro std
nbh voro vol mean
nbh voro vol std
nbh n neigh mean
nbh n neigh std
nbh centr off mean
nbh centr off std
msd 10
msd 20
msd 30
feature value
i

Supplementary Figure 4: Organisational feature definition, clustering and distributions

a: Illustration of multi-scale kernel density estimation. Density is measured for each nucleus at three radii (10, 20, and 30 µm). For the smallest radius, density is computed as the number of nuclei within a 10 µm sphere divided by the sphere's volume. For larger radii, a spherical shell (10–20 µm or 20–30 µm) is used, and the nucleus count is divided by the shell volume for normalisation. **b:** Correlation of density measurements between radii, using N = 34 samples and n = 4,080,605 cells. Selected radii show <50% correlation. The smallest radius was chosen to avoid 0-count bias and 0 μm^{-3} densities. **c:** Organisational features derived from 3D restricted Voronoi diagrams. For each point, the Voronoi cell volume, mean inverse distance to neighbours, neighbour count, and offset between seed and centroid are measured. **d:** Local variability of Voronoi features is assessed by computing the mean and standard deviation within each point's neighbourhood. These, together with single-cell features, describe local organisation explicitly. **e**: Hierarchical agglomerative clustering (Ward linkage on z-scored features, Euclidean distance) groups features into three classes based on spatial autocorrelation. Density and anisotropy features show anti-correlation, indicating some co-linearity. Abbreviations: voro = Voronoi, vol = volume, nbh = neighbourhood, cntr off = centroid offset, msd = multi-scale density, neigh = neighbours. **f:** Global histograms of z-scored feature values lie within ±2 SD of the mean. Density and anisotropy histograms differ, indicating they provide distinct information despite co-linearity. Histogram density estimated via Gaussian kernel with bandwidth set by Scott's rule[6], scaled by 1.4 to suppress artefacts (N = 34, n = 4,080,605). **g:** Mean contribution of each feature to the first two principal components, averaged across 10 replicates of 100,000 randomly sampled points. **h:** Spatial distributions of z-scored feature values projected across embryos. Features within the same class highlight similar, but non-identical, anatomical regions, indicating unique informational content. **i:** Feature values projected on a t-SNE embedding of the organisational space (100×100 hex bins). Intra-class distributions are locally similar. Bins with fewer than n/30,000 cells are excluded to minimise edge effects (N = 34, n = 4,080,605). Source data are provided.

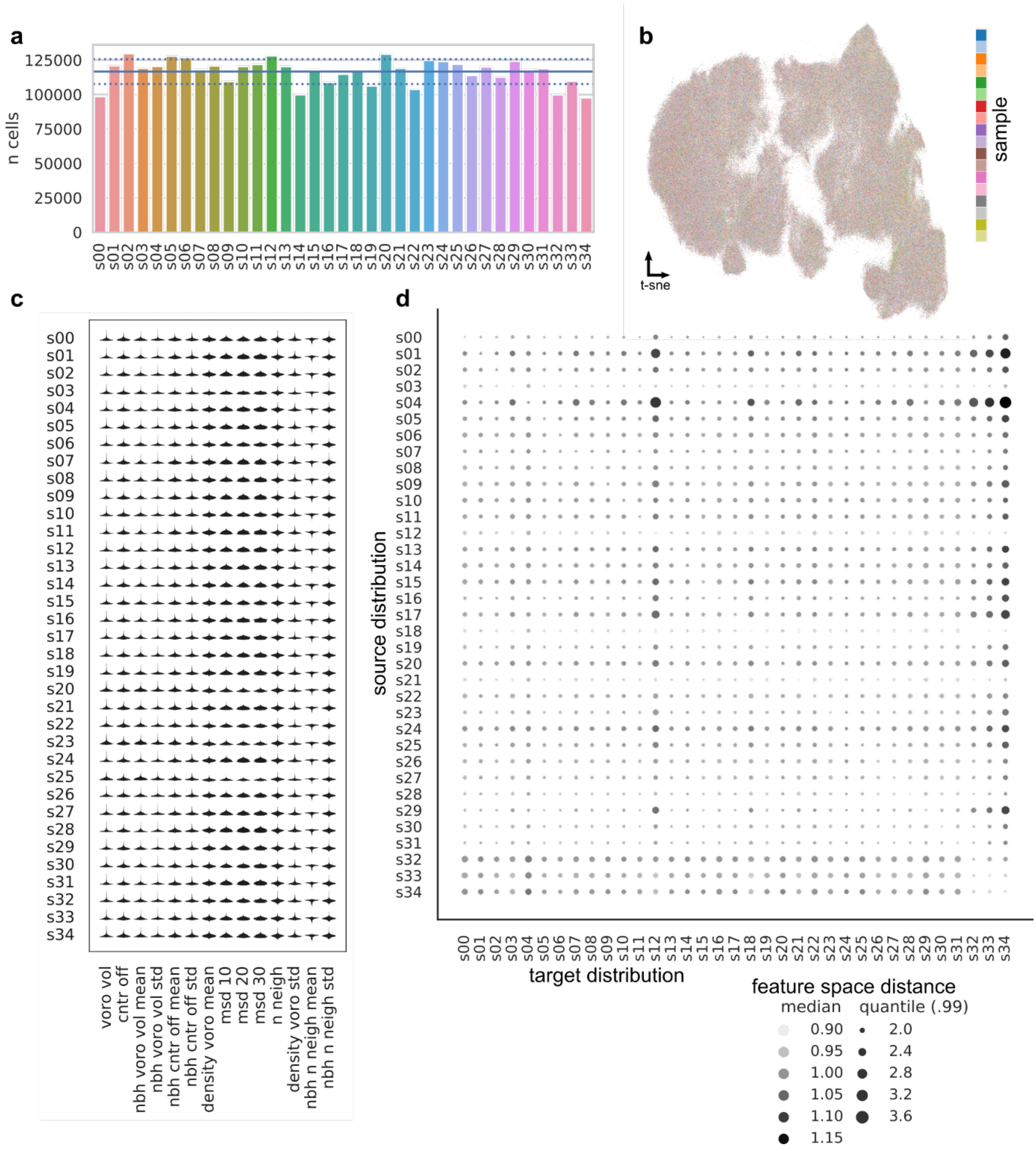

Supplementary Figure 5: Feature distributions of samples are aligned

a: Number of nuclei are similar between samples. The mean number of cells per sample at 48 hpf was 116,600 ± 9,000 (7.72%); solid line denotes mean, dashed line mean ± standard deviation. **b**: Organisational feature space is well mixed. Individual samples are coloured individually, however, no region of the t-SNE embedding stands out with a single colour, indicating that, overall, the used samples are similar in terms of their organisational feature distributions. **c**: Distributions of organisational feature values are qualitatively similar between samples. **d**: Distance between samples in organisational feature space. Shown are the distance between single points in the source distribution (y-axis) and their nearest neighbour in the target distribution (x-axis) as total median and the mean of the 1% most distant points. Distance metric: Euclidean distance in 14-dimensional space. Individual samples are labelled sXX (e.g. s01). N = 34 samples and n = 4,080,605 cells. Source data are provided as a Source Data file.

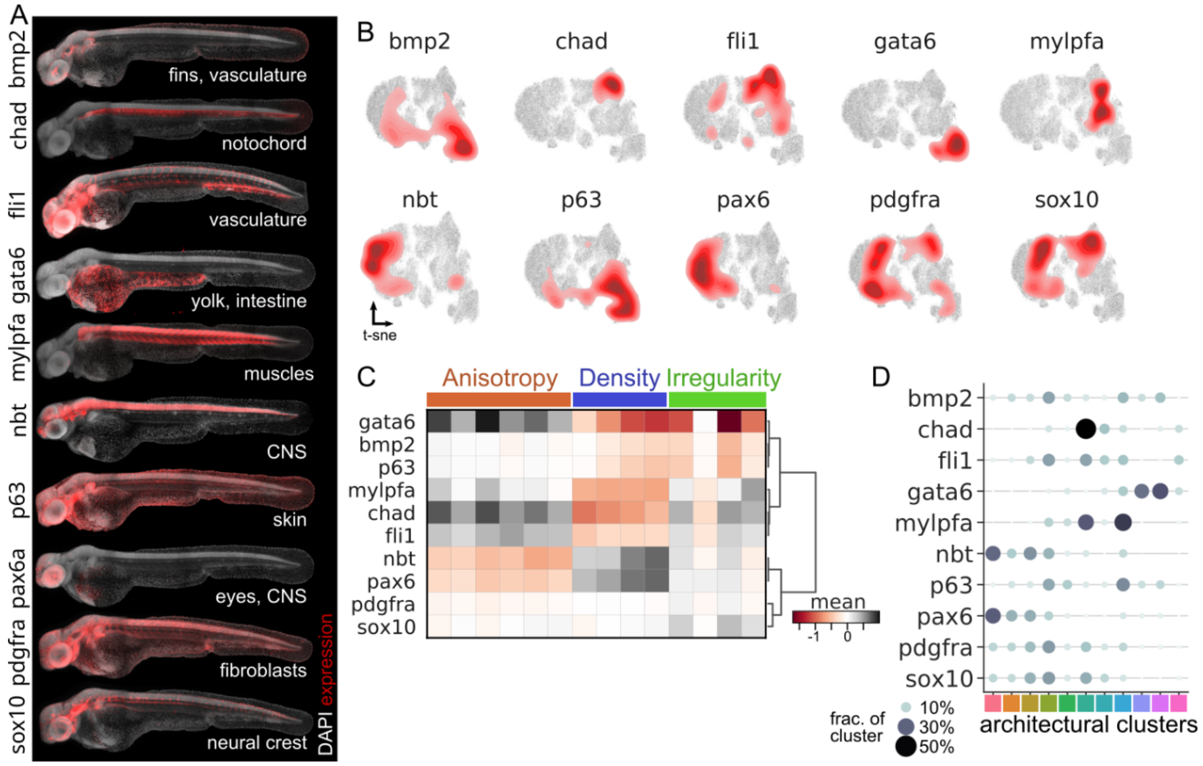

Supplementary Figure 6: Tissue/cell type specific markers exhibit distinct tissue organisational profiles.

A: Spatial distributions of the labelled cells. Maximum intensity projections of in toto multi-view fused light sheet images of 48 hpf zebrafish embryos. Most genes were labelled via HCR RNA FISH, with the exception of nbt and fli1 for which the transgenic fishlines Tg(NBT:dsRed) and Tg(fli1:GFP) were used. Markers were pairwise multiplexed, e.g., chad and mylpfa were stained in the same sample and were imaged in different fluorescent channels. CNS: central nervous system. B: Mapping of marker positive cells onto a t-SNE of the organisational feature space. Red regions represent the density of marker positive cells; grey points are non-expressing and are shown for reference. Density of positive points on embeddings was estimated using a Gaussian kernel estimation with the bandwidth selected via Scott's rule[6]. C: Mean organisational feature profiles of marker positive domains. Clustering based on feature similarity links related tissues such as nbt and pax6. Hierarchical agglomerative clustering was performed using Ward linkage[7] and Euclidean distance on z-scored feature values. D: Marker positive domains are composed of unique distributions of organisational motifs (clusters). Note that the distribution of motifs adds to 100% for each marker independently of its total abundance and that the distributions do not add to 100% for each motif. Sample numbers: bmp2 N=3 (samples), n=25,158 (total cells); chad N=3, n=7;545, fli1 N=3, n=119,514; gata6 N=3, n=18,591; mylpfa N=3, n=31,633, nbt N=2, n=50,844; p63 N=3, n=82,698; pax6 N=3, n=26,857; pdgfra N=3, n=61,355; sox10 N=3, n=28,888. Source data are provided as a Source Data file.

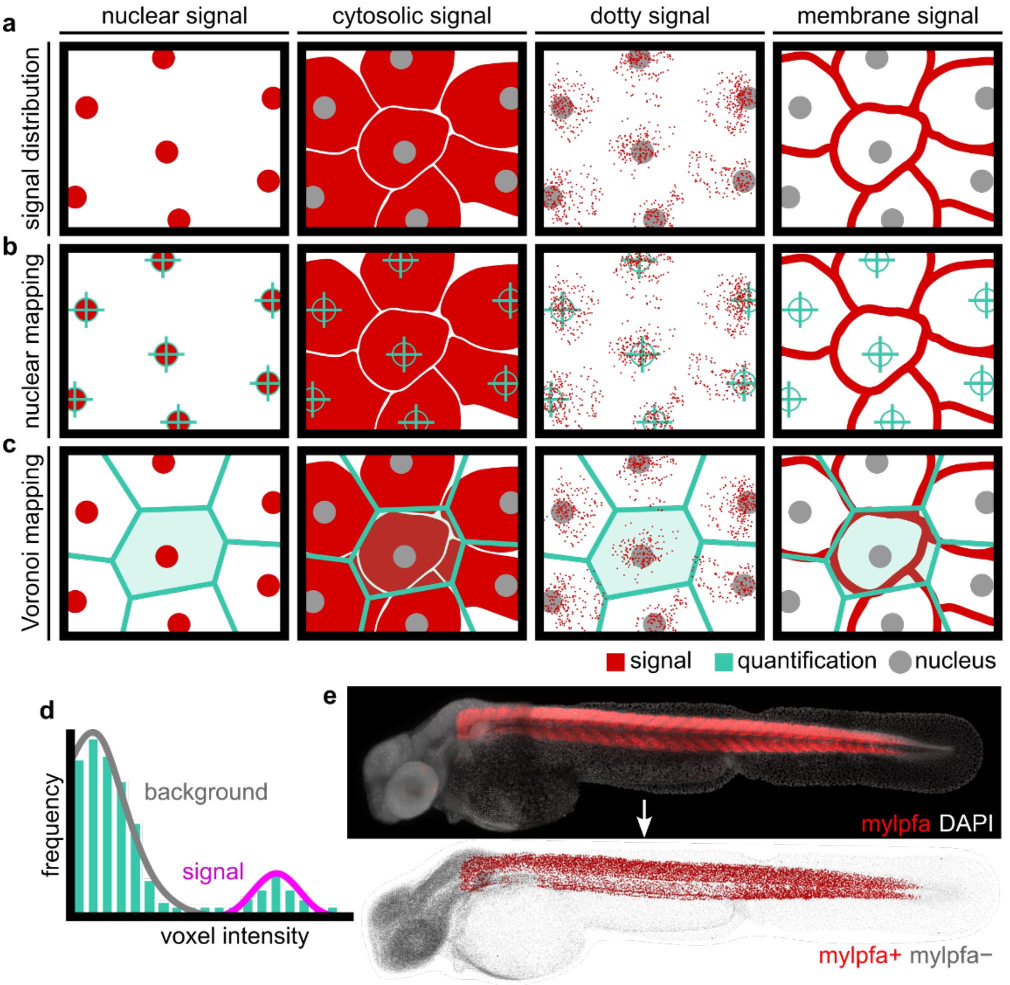

Supplementary Figure 7: Illustration of intensity classification

a: Different localisations of signals in cells encountered in this study. Depending on the labelling technique or the used transgenic fishlines, localisation of the fluorescent signal within the cell was often externally determined. **b**: Nuclear mapping uses the location of the segmented nuclei to integrate intensities within a spherical region. The radius of that region is a free parameter and needs to be adjusted to the size of the measured object. **c**: Integration within individual Voronoi cells enabled intensity measurements in a larger area in proximity to individual cells. Subsequently, individual pixels were analysed to determine whether a cell was marker positive. **d**: Illustration of the histogram-based pixel classification for the Voronoi integration scheme via Gaussian noise model. Two Gaussian distributions were fitted to the histogram. One captured the noise and one the signal, enabling subsequent analyses to classify cells as positive/negative via global thresholds. **e**: Transformation of an in toto light sheet image of a 48 hpf zebrafish embryo labelled with DAPI and HCR RNA FISH (spot-like pattern) against the muscle specific gene mylpfa into a point cloud representation with mylpfa positive cells highlighted. Maximum intensity projections.

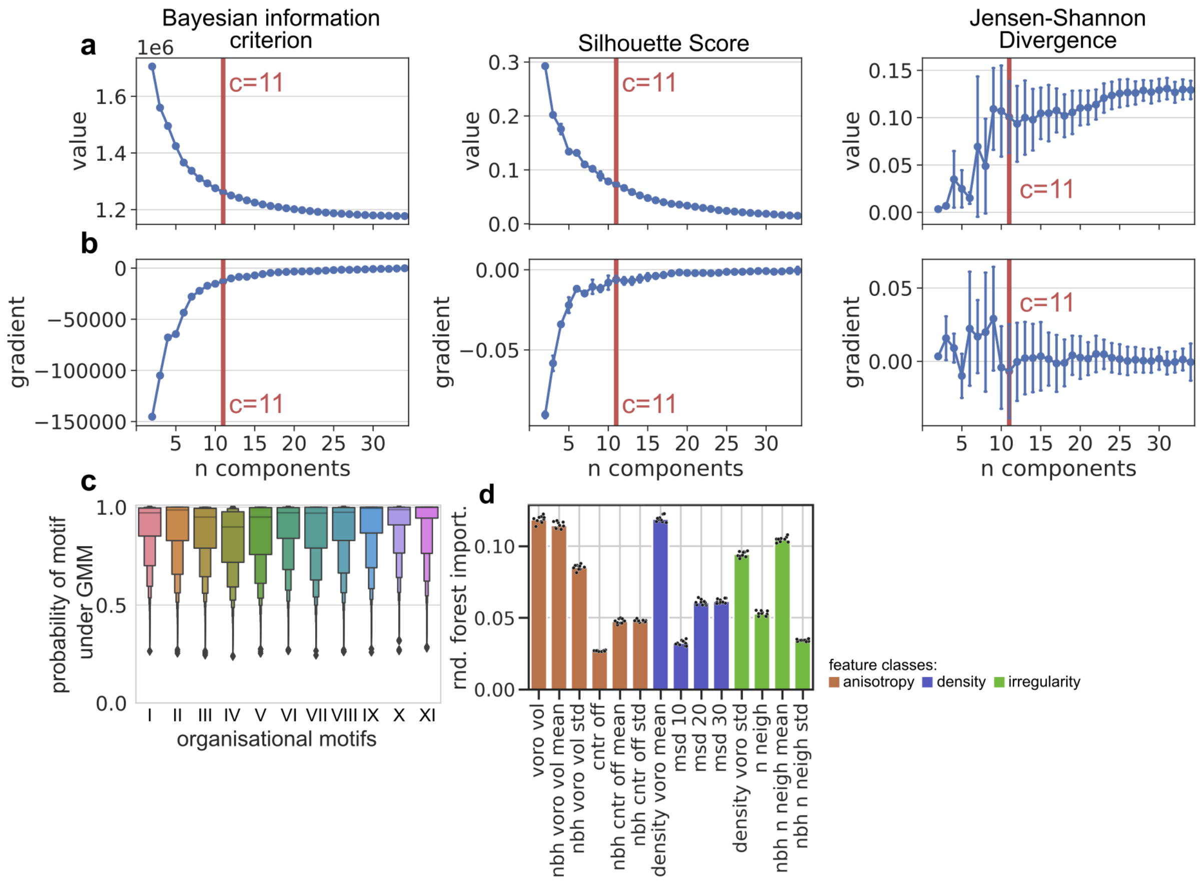

Supplementary Figure 8: Determination of number of organisational motifs

Organisational motifs are determined via Gaussian mixture model (GMM) based on individual cell's organisational feature profiles. To determine the appropriate number of GMM components for the organisational data of 48 hpf zebrafish embryos (N=34, n=4,080,605), GMMs with between 2 and 34 components were generated from 1% randomly selected nuclei and the performance of each GMM was evaluated using three metrics: Bayesian information criterion (BIC), Silhouette score (SilS) and Jensen-Shannon Divergence (JSD) (see Suppl. Note 3 for details on the used metrics). This process was repeated 100 times to estimate variability. **a** shows the performance of the three metrics and **b** the gradient of these metrics for consecutive number of components. Error bars denote standard deviation of the mean. Using standard criteria, the number of components is determined to 11[8,9]. For this number of components, the average probability of correct component assignment for each point and component was 97% (median) and never lower than 90% (motif IV), indicating that the identified number of components appropriately models the distribution of nuclei in organisational feature space (**c**). Letter value plot (c) indicates median (line) and multiple quantiles (boxen). **d:** Individual organisational features contribute to the identification of the organisational motifs. Importance of organisational features for clustering is measured by training a random forest classifier on the organisational features and motif labels; importance of individual features is then assessed through evaluating its utilisation in the classifier. Values indicate the fraction of contribution of each feature to the clustering. Shown are mean importance scores for 10 independent evaluations of 100,000 randomly selected nuclei. Source data are provided as a Source Data file.

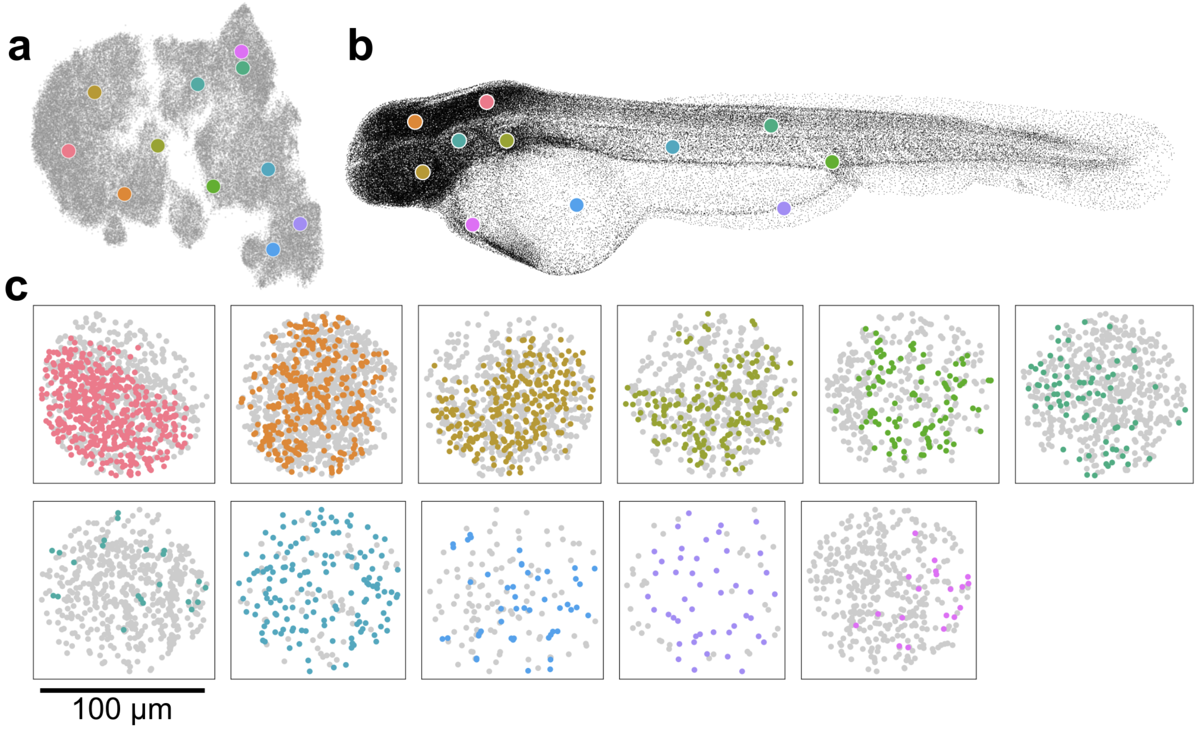

Supplementary Figure 9: Illustration of organisational motifs

Local distribution of nuclei for individual organisational motifs. **a**: Localisation of cells with motif-average feature profile (see Fig. 2 a) on feature space embedding. **b**: Same points projected on a segmented embryo. **c**: Local distribution of motif-associated nuclei (colour) and other nuclei (grey). Shown are z-flattened distributions of a sphere with 50 µm radius centred around the points shown in **a** and **b**.

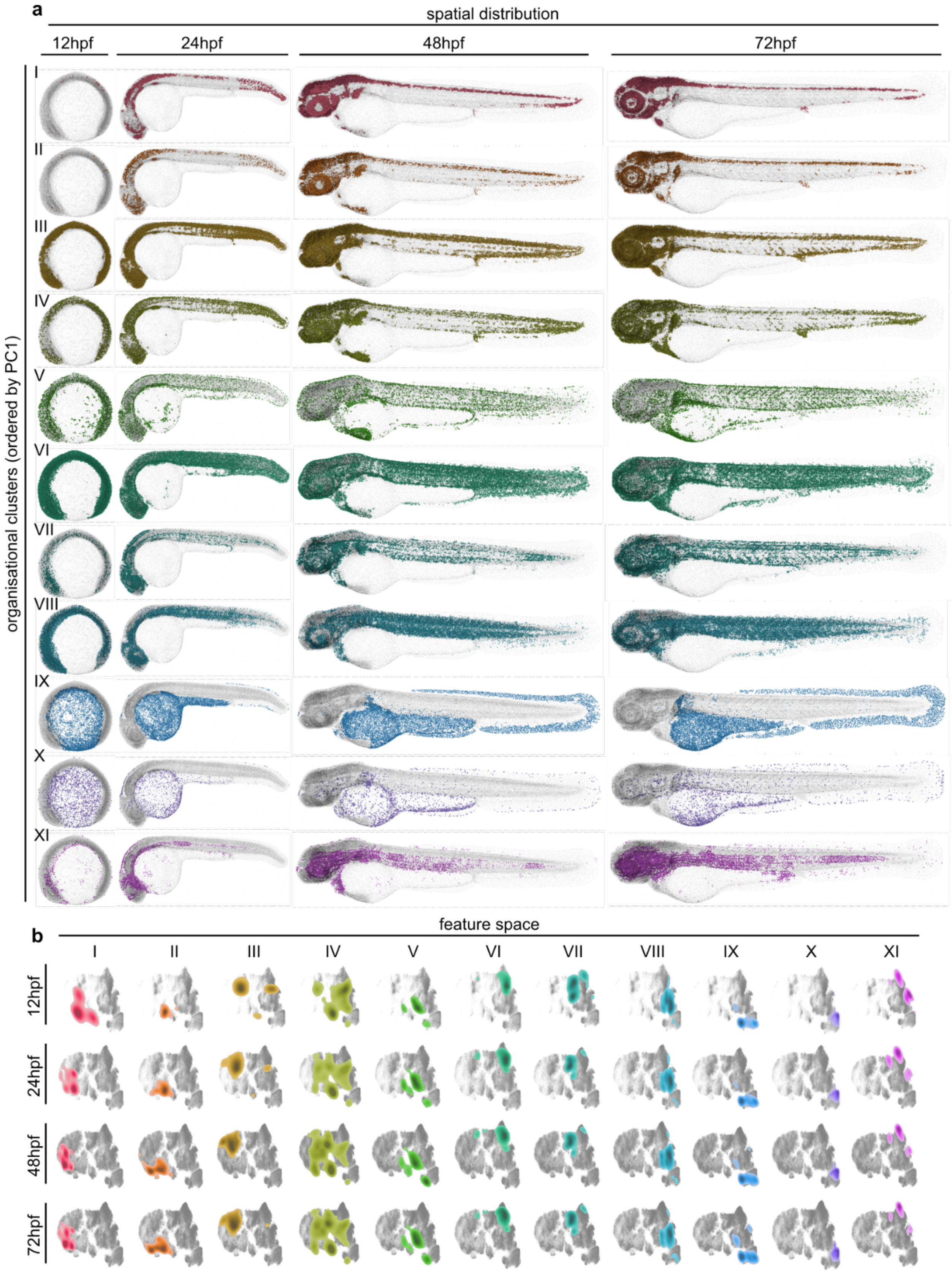
a
spatial distribution
12hpf
24hpf
48hpf
72hpf
organisational clusters (ordered by PC1)
I
II
III
IV
V
VI
VII
VIII
IX
X
XI
b
feature space
I
II
III
IV
V
VI
VII
VIII
IX
X
XI
12hpf
24hpf
48hpf
72hpf

Supplementary Figure 10: Distribution of organisational motifs

a: Spatial distributions of organisational motifs at different developmental stages. Motifs map to compact, connected regions on the samples that qualitatively encompass different anatomical regions. This highlights the organisational similarity between unrelated tissues. Note that no explicit point coordinates were used during the generation of the motifs. Motifs are ordered by the level of the average first principal component across the whole feature set. **b**: Individual clusters at different time points mapped onto a temporally joint feature space. Note that the underlying organisational feature space (grey regions) develops over time, limiting the extents of some motifs. Density of positive points on embeddings was estimated using a Gaussian kernel estimation with the bandwidth selected via Scott's rule (Scott, 1992); density is not normalised to total cell number. Grey dots represent cells outside a specific motif. N=16, n=1,698,230

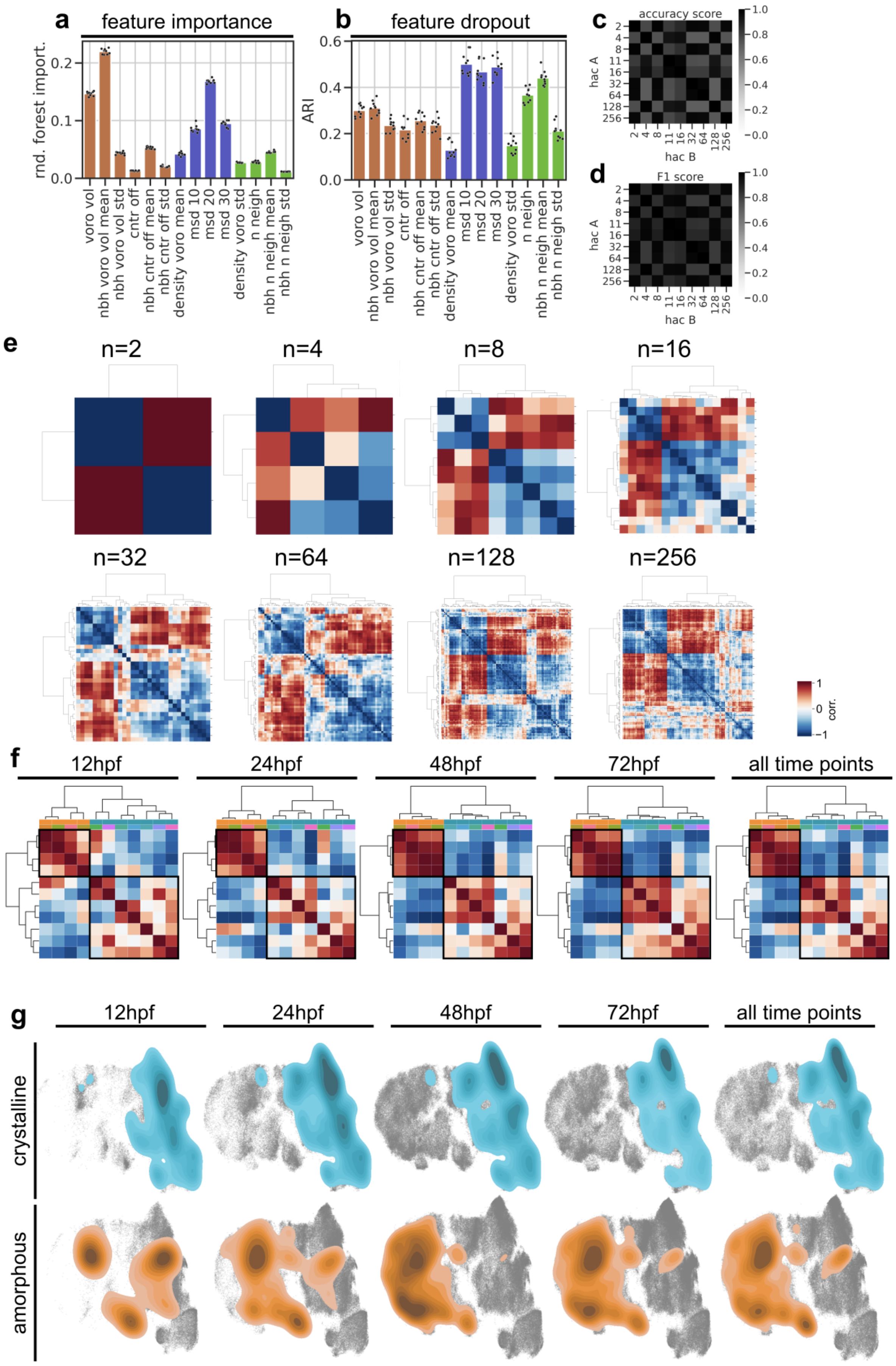

a
feature importance
rnd. forest import.
b
feature dropout
ARI
voro vol
nbh voro vol mean
nbh voro vol std
cntr off
nbh cntr off mean
nbh cntr off std
density voro mean
msd 10
msd 20
msd 30
density voro std
n neigh
nbh n neigh mean
nbh n neigh std
c
accuracy score
hac A
hac B
d
F1 score
e
n=2
n=4
n=8
n=16
n=32
n=64
n=128
n=256
corr.
f
12hpf
24hpf
48hpf
72hpf
all time points
g
crystalline
amorphous

Supplementary Figure 11: Organisational archetypes are independent of motif number and exist at all analysed time points

a, b: Feature importance for identifying organisational archetypes. **a**: Density and anisotropy features contribute most to class separation. Importance of organisational features for clustering is measured by training a random forest classifier on the organisational features and motif labels; importance of individual features is then assessed through evaluating its utilisation in the classifier. Values indicate the fraction of contribution of each feature to the clustering. Mean importance scores for 10 independent evaluations of 100,000 randomly selected nuclei. **b**: Feature dropout analysis shows clustering performance decreases when omitting individual features. Classification into organisational archetypes was performed on data lacking individual features. Resulting classifications were compared to the classification using the full feature set. Adjusted Rand Index (ARI) measures clustering similarity any feature results in decreased ARI. **c**-**e**: Archetype identification is independent of the number of GMM components used for initial clustering. While more GMM components reveal additional substructures in the data, all analysed GMM numbers exhibit a similar fundamental bipartition (**c**) and assign individual nuclei consistently to the same archetype (**d, e**). Accuracy is calculated as $Accuracy = (TP + TN)/(TP + TN + FP + FN)$ with TP (true positive), TN (true negative), FP (false positive), FN (false negative). F1 score is defined as $F1 = 2 \cdot (precision \cdot recall)/(precision + recall)$ with $precision = TP/(TP + FP)$ and $recall = TP/(TP + FN)$. **f**: Two organisational archetypes are present at every time point individually. Intra-cluster correlation and inter-cluster anticorrelation increase over time. Similarity of organisational motifs based on mean organisational features per motif. Joint analysis of all time points. Hierarchical clustering performed on z-scored feature values using Ward linkage (Ward, 1963) and Euclidean distances. Colours on upper two rows indicate archetype and motif association respectively. **g**: The organisational archetypes map to opposite sides of the organisational feature space embedding and show little overlap at every time point individually. Density of positive points on embeddings was estimated using a Gaussian kernel estimation with the bandwidth selected via Scott's rule (Scott, 1992). t-SNE embedding of organisational feature space. N=16 (4 per time point), n=1,698,230. Colormap in **e** and **f** indicates pairwise correlation of organisational features between organisational motifs (red=1, white=0, blue=-1). Source data are provided as a Source Data file.

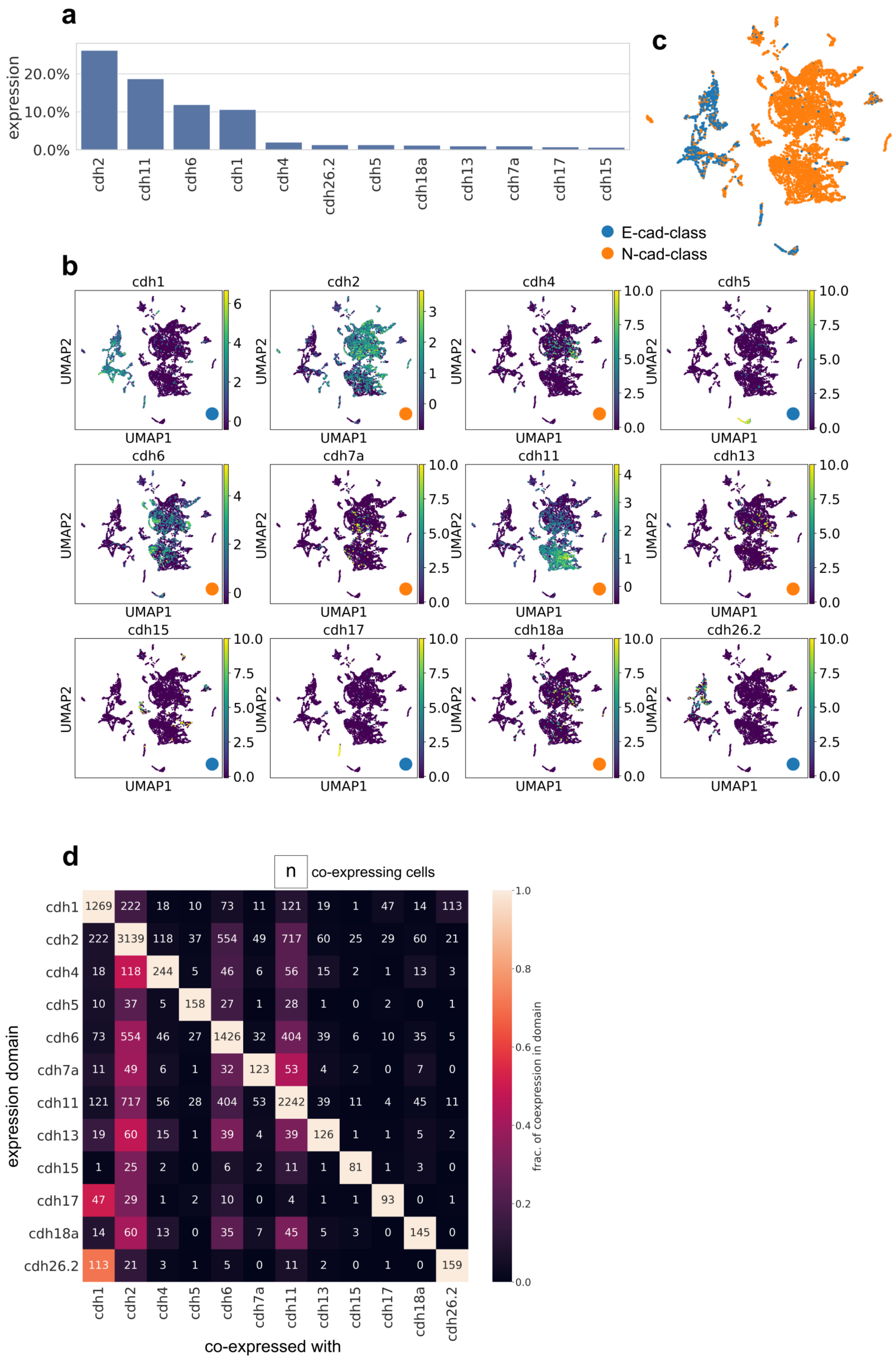
a
expression
20.0%
10.0%
0.0%
cdh2 cdh11 cdh6 cdh1 cdh4 cdh26.2 cdh5 cdh18a cdh13 cdh7a cdh17 cdh15
c
E-cad-class
N-cad-class
b
cdh1 cdh2 cdh4 cdh5
cdh6 cdh7a cdh11 cdh13
cdh15 cdh17 cdh18a cdh26.2
UMAP1
UMAP2
d
n co-expressing cells
expression domain
co-expressed with
frac. of coexpression in domain
cdh1 1269 222 18 10 73 11 121 19 1 47 14 113
cdh2 222 3139 118 37 554 49 717 60 25 29 60 21
cdh4 18 118 244 5 46 6 56 15 2 1 13 3
cdh5 10 37 5 158 27 1 28 1 0 2 0 1
cdh6 73 554 46 27 1426 32 404 39 6 10 35 5
cdh7a 11 49 6 1 32 123 53 4 2 0 7 0
cdh11 121 717 56 28 404 53 2242 39 11 4 45 11
cdh13 19 60 15 1 39 4 39 126 1 1 5 2
cdh15 1 25 2 0 6 2 11 1 81 1 3 0
cdh17 47 29 1 2 10 0 4 1 1 93 0 1
cdh18a 14 60 13 0 35 7 45 5 3 0 145 0
cdh26.2 113 21 3 1 5 0 11 2 0 1 0 159
cdh1 cdh2 cdh4 cdh5 cdh6 cdh7a cdh11 cdh13 cdh15 cdh17 cdh18a cdh26.2

Supplementary Figure 12: Cadherin expression in scRNAseq analysis exhibits bipartitioning which is not explained by single cell co-expression.

a: Cadherin expressing cells that exceed 1% of all cells in the dataset were selected for analysis. cdh2, cdh11, cdh6, and cdh1 are more broadly expressed than the other domains. Note that individual cells can express multiple cadherins and hence, the total proportion does not add up to 100%. Data from one dataset; bars indicate singular measurement. **b**: Individual cadherin domains localise to specific regions of transcriptome embedding. Domains of the E-cadherin-like class (blue) localise to regions that are distinct within the class and between classes, while domains of the N-cadherin-like class co-localise within the class. Scale bars denote the number of transcripts detected per cell. **c**: Localisation of E-cadherin-class and N-cadherin-class cells in transcriptional feature space embedding. Cells E- and N-cadherin-class domains form distinct and predominantly mutually exclusive regions, indicating different transcriptional states. Only cadherin expressing cells are shown. Cells were classified as expressing, if at least one cadherin transcript of was detected. UMAP embedding was calculated on the selected subset using the first 50 principal components of all transcripts according to (Kobak and Berens, 2019). **d**: Pairwise co-expression of cadherins in scRNAseq dataset. The diagonal entries show the number of cells per cadherin expression domain. Numbers in the rest of the graph indicate the number of cells co-expressing two cadherins. Colouring of the boxes indicates the percentage of cells of one cadherin expression domain (y-axis) co-expressing another cadherin (x-axis). E.g., the orange entry on the bottom left shows that 113 cells co-express cdh26.2 and cdh1; the orange colour reports that 71% of cdh26.2 expressing cells co-express cdh1. The broadest overlap was found between cdh26.2 and cdh1, with 71% of cdh26.2 expressing cells also expressing cdh1. Apart from this overlap, no co-expression exceeded 50% and most cadherins were not co-expressed at significant levels. Importantly, most cdh2 expressing cells do not co-express another cadherin. Data from[10], 48 hpf, n=12,005 cells. Source data are provided as a Source Data file.

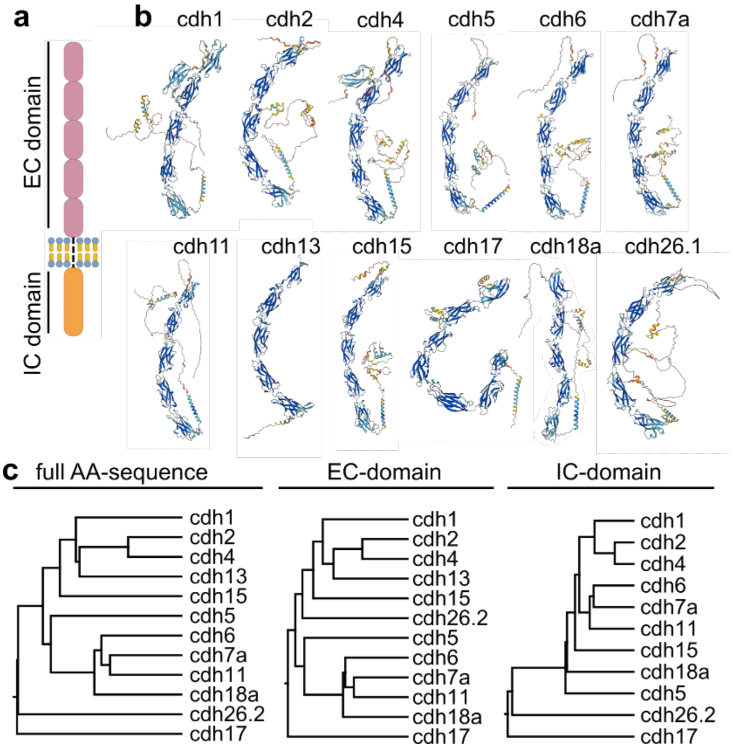

Supplementary Figure 13: Organisational classification is not predicted by protein similarity

a: Illustration of a generic cadherin protein structure. The extracellular (EC) domain consists of several (commonly five) repeating cadherin domains, while the intracellular (IC) domain has no general stereotypic structure. **b**: Predicted protein structures of the analysed cadherins. Most structures are qualitatively similar and recapitulate the generic model. The most obvious exceptions are cdh1 and cdh17 with six and seven EC repeats. Structures predicted by Alphafold (DB version 2022-11-01)[11,12]. **c**: Similarity of cadherin amino acid sequences does not recapitulate clustering based on transcriptional or organisational features. Alignments are based on the cadherin's full-length sequences, their extracellular domains only and their intracellular domains only. Trees built using BLOSUM85 cost matrix, Jukes-Cantor genetic distance model and UPGMA via geneious v2022.0[13].

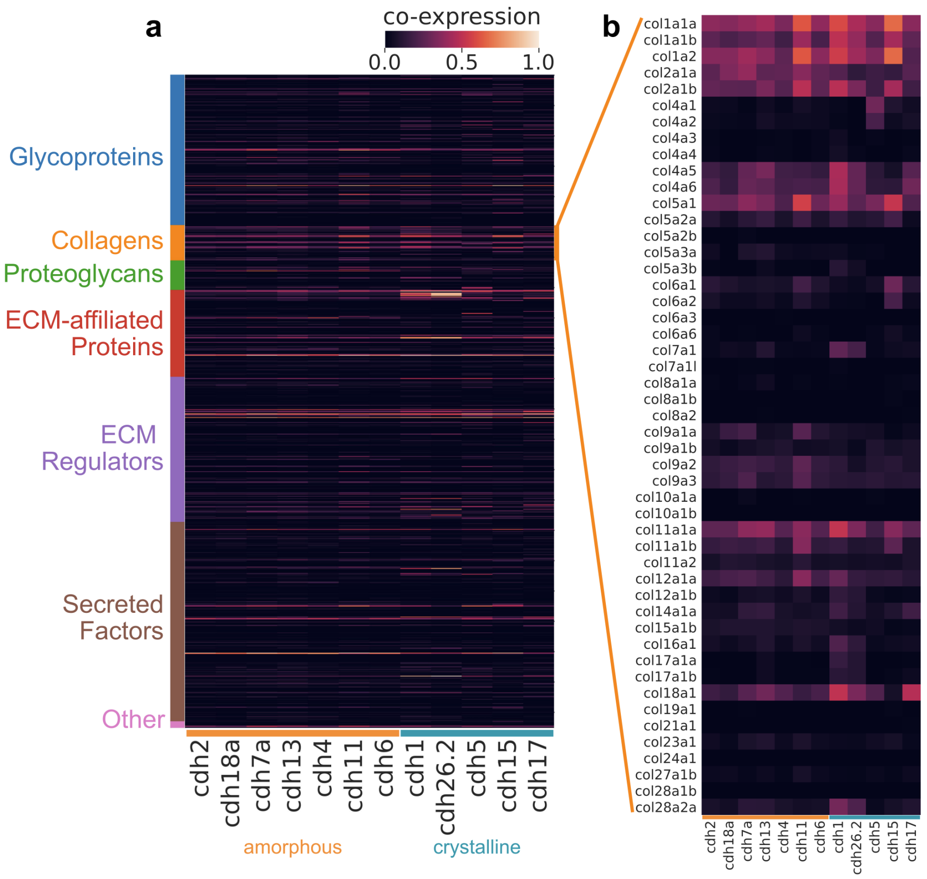

Supplementary Figure 14: Expression of ECM associated genes exhibits heterogeneous association with cadherins.

a: Co-expression of matrisome components (Nauroy et al., 2018) with major cadherins at 2 dpf reveals heterogenous association. Co-expression is calculated as fraction of cells expressing a specific matrisome component of cells expressing a specific cadherin. scRNAseq data from[10], n=12,005 cells. **b**: Focussed analysis of collagens further demonstrates heterogenous association with cadherins. Source data are provided as a Source Data file.

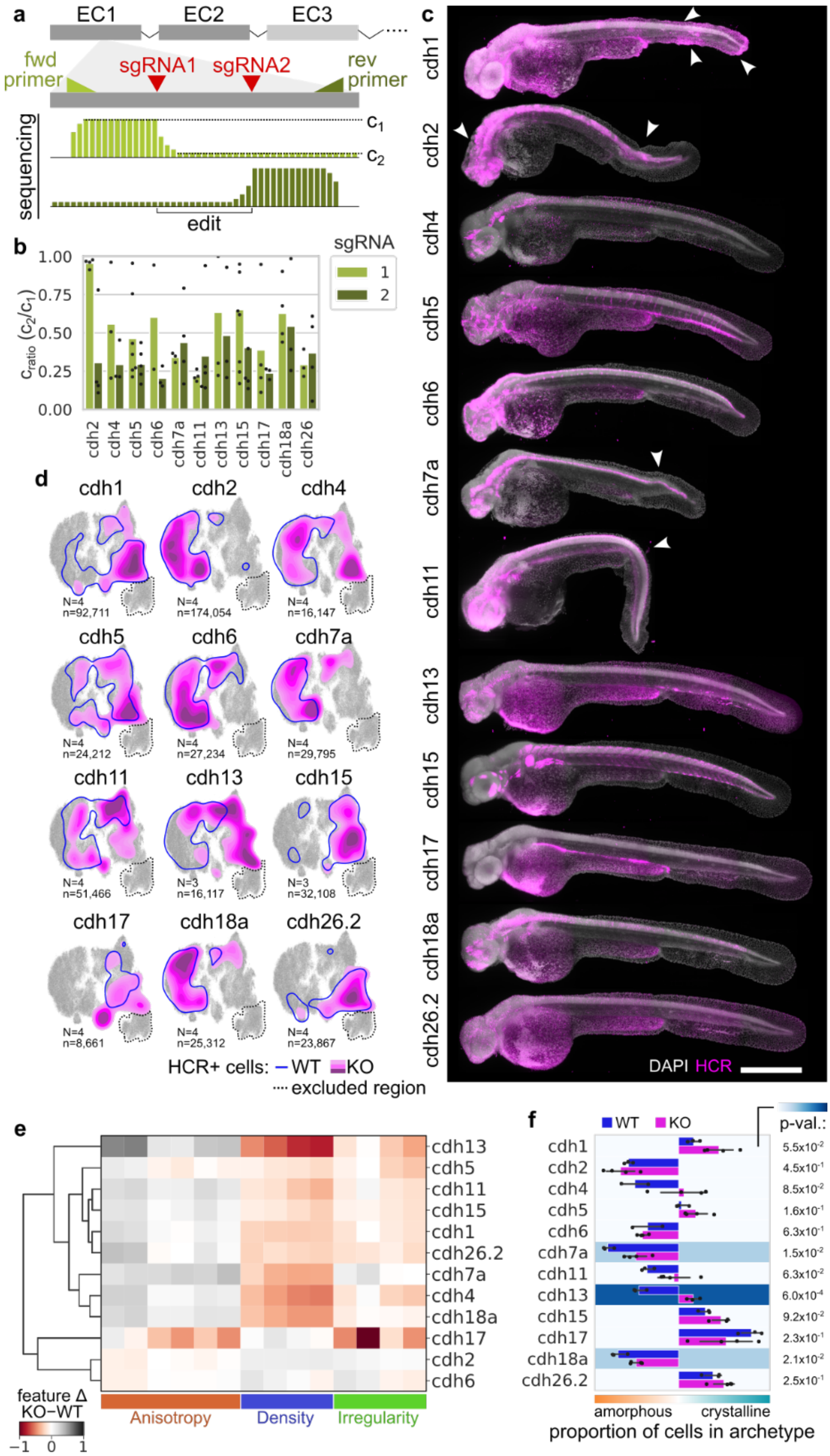

a
EC1
EC2
EC3
fwd primer
sgRNA1
sgRNA2
rev primer
sequencing
c1
c2
edit
b
sgRNA
1
2
1.00
0.75
0.50
0.25
0.00
c_ratio (c2/c1)
cdh2 cdh4 cdh5 cdh6 cdh7a cdh11 cdh13 cdh15 cdh17 cdh18a cdh26
c
cdh1 cdh2 cdh4 cdh5 cdh6 cdh7a cdh11 cdh13 cdh15 cdh17 cdh18a cdh26.2
DAPI HCR
d
cdh1 N=4 n=92,711
cdh2 N=4 n=174,054
cdh4 N=4 n=16,147
cdh5 N=4 n=24,212
cdh6 N=4 n=27,234
cdh7a N=4 n=29,795
cdh11 N=4 n=51,466
cdh13 N=3 n=16,117
cdh15 N=3 n=32,108
cdh17 N=4 n=8,661
cdh18a N=4 n=25,312
cdh26.2 N=4 n=23,867
HCR+ cells: WT KO
excluded region
e
cdh13 cdh5 cdh11 cdh15 cdh1 cdh26.2 cdh7a cdh4 cdh18a cdh17 cdh2 cdh6
feature Δ KO−WT
−1 0 1
Anisotropy
Density
Irregularity
f
WT KO
p-val.:
cdh1 5.5x10^-2
cdh2 4.5x10^-1
cdh4 8.5x10^-2
cdh5 1.6x10^-1
cdh6 6.3x10^-1
cdh7a 1.5x10^-2
cdh11 6.3x10^-2
cdh13 6.0x10^-4
cdh15 9.2x10^-2
cdh17 2.3x10^-1
cdh18a 2.1x10^-2
cdh26.2 2.5x10^-1
amorphous
crystalline
proportion of cells in archetype

Supplementary Figure 15: Systematic knockout of individual cadherins using F0 CRISPR/Cas9

a: Transient 'null mutants' are generated by targeting two sites of the target cadherin's EC1 or EC2 domain with sgRNAs. Successful knockout (frame shift or large deletion) was confirmed via Sanger sequencing across the sgRNAs' homology region. A drop in read quality in the chromatogram following a sgRNA target site indicated a successful edit. c_1: chromatogram level pre-sgRNA; c_2: chromatogram level post-sgRNA; in direction of the sequencing primer. **b**: Transient knockout of individual cadherins was efficient. c_{ratio} (c_2 / c_1) measures the drop in sequencing quality following a sgRNA cut site. Values range from 1 (no edit) to 0 (no signal post site). sgRNA1 of cdh2 did not cause a genome edit. All other sgRNAs edited the genome on average. Whole-embryo cdh1 KO was lethal; consequently, verification via sequencing was not possible; cdh1 was knocked out in a mosaic fashion by 8-cell stage injection. **c**: Deletion of individual cadherins causes gross phenotypes for cdh1, cdh2 cdh7a and cdh11. The knockout of the other cadherins did not induce obvious embryo-wide effects. Maximum intensity projections of in toto light sheet images. Nuclei are stained with DAPI and the transcripts of the target cadherins are visualised using HCR RNA FISH. Arrowheads indicate phenotypic regions. **d**: Knockout of cadherin induces organisational change in transcript-expressing cells. Distributions of cadherin transcript positive cells on t-SNE representation of organisational feature space. Solid magenta regions are cells from the knockout condition, blue outline shows corresponding regions in wildtype samples (see Figure 3), grey regions are non-expressing cells for reference. Regions within the dotted line (cells on yolk) were not included in the analysis due to unspecific labelling of the yolk. **e**: Feature difference between cadherin transcript positive cells in knockout and wildtype. Most domains exhibit a notable shift in organisational features. **f**: Organisational archetype association of cadherin transcript positive cells in wildtype and knockout. Background colour indicates the difference between mean archetype proportions in knockout and wildtype. Most cadherin knockouts organise more crystalline. Error bars indicate 95% confidence interval. Sample number as indicated in D (KO) and Figure 3 (WT). Source data are provided as a Source Data file.

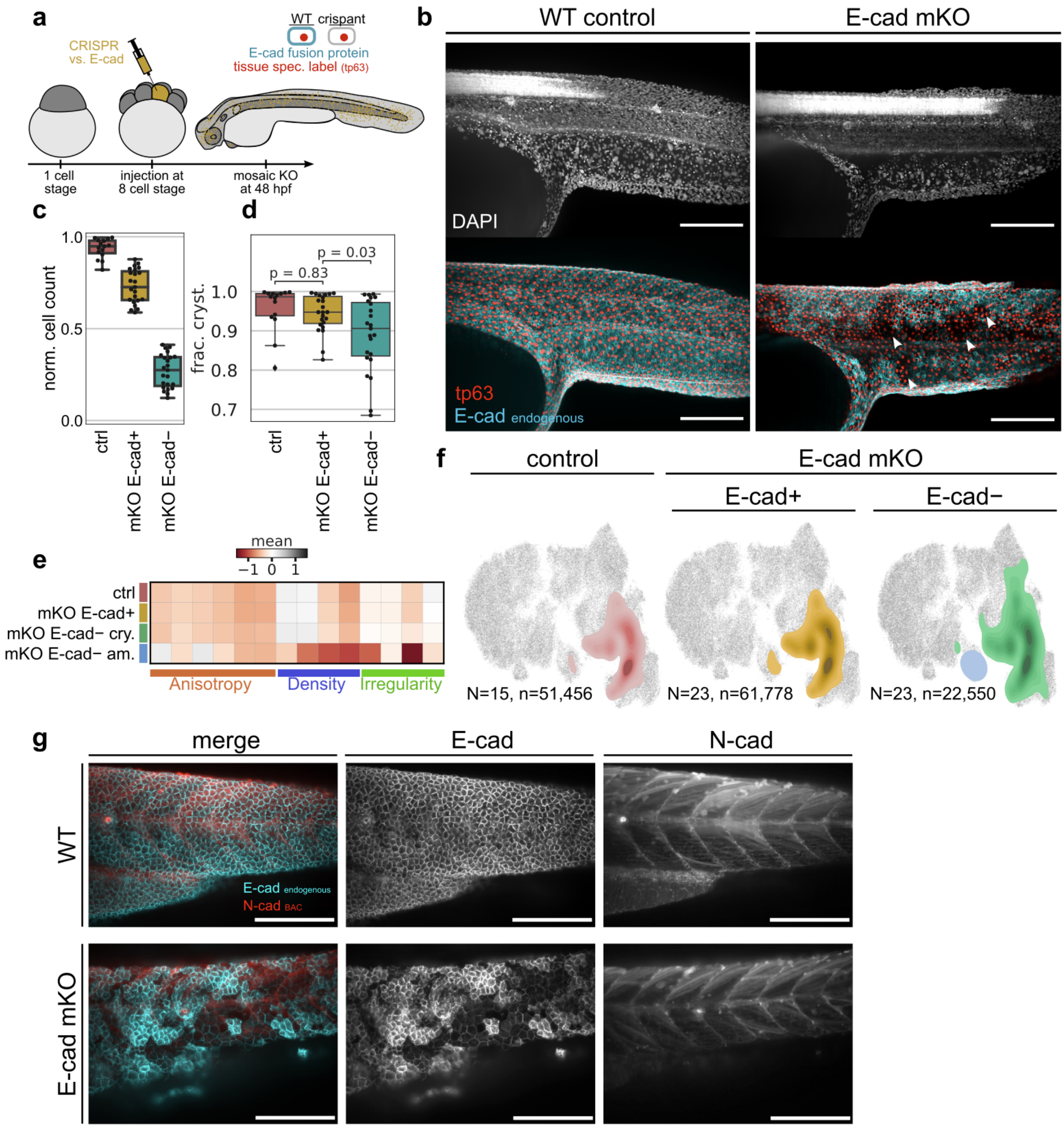
a
CRISPR vs. E-cad
WT crispant
E-cad fusion protein
tissue spec. label (tp63)
1 cell stage
injection at 8 cell stage
mosaic KO at 48 hpf
b
WT control
E-cad mKO
DAPI
tp63
E-cad endogenous
c
norm. cell count
1.0
0.5
0.0
ctrl
mKO E-cad+
mKO E-cad−
d
frac. cryst.
p = 0.03
p = 0.83
1.0
0.9
0.8
0.7
ctrl
mKO E-cad+
mKO E-cad−
e
mean
−1 0 1
ctrl
mKO E-cad+
mKO E-cad− cry.
mKO E-cad− am.
Anisotropy
Density
Irregularity
f
control
E-cad mKO
E-cad+
E-cad−
N=15, n=51,456
N=23, n=61,778
N=23, n=22,550
g
merge
E-cad
N-cad
WT
E-cad mKO
E-cad endogenous
N-cad BAC

Supplementary Figure 16: Crystalline basal skin organisation shifts slightly but significantly towards amorphous in the absence of E-cadherin

a: Late injection generates identifiable mosaic knockout (mKO) of E-cadherin. CRISPR mediated mKO of E-cadherin was generated by injecting sgRNAs+Cas9 at the 8-cell stage of zebrafish development. Samples had a transgenic fluorescent label for cells in the basal skin layer (tp63) and an endogenous E-cadherin fusion protein (TgCRISPR(cdh1-mLanYFP)). WT cells express both tp63 and E-cadherin; crispant cells are positive for tp63 and negative for E-cadherin. **b**: E-cadherin mKO is successful and induces clonal loss of E-cadherin in the embryonic skin. Dorsal view (shallow maximum intensity projection) of the trunk of a 48hpf zebrafish embryo. tp63 expressing cells co-express E-cadherin in WT control but not generally in N-cadherin mKO. tp63 proteins levels were visualised using antibody staining. Arrowheads indicate regions of clonal E-cadherin knockout. **c**: E-cadherin mKO induces loss of E-cadherin in 27.0 ± 9.0 % of basal skin cells. **d**: Fraction of amorphously organised cells increases slightly but significantly in E-cad mKO cells over E-cad+ cells; the latter organise not significantly different from E-cad+ cells of control embryos. **e**: Shift towards amorphous organisation is driven by a decrease in cell density and in regularity. **f**: Projection of p63+ cells onto organisational feature space representation reveals slight archetypal shift towards amorphous organisation exclusive in E-cadherin mKO cells. Organisation of segmented nuclei was quantified per sample and subsequently aligned with the in toto organisational feature space. N: number of samples, n: total number of analysed cells. **g**: N-cadherin does not compensate for the loss of E-cadherin in mKOs. Arrowheads indicate patches of mosaically lost E-cadherin expression that do not upregulate N-cadherin. Box plots show median (centre), interquartile range (box), and 1.5× IQR (whiskers). All scale bars 200 µm. Source data are provided as a Source Data file.

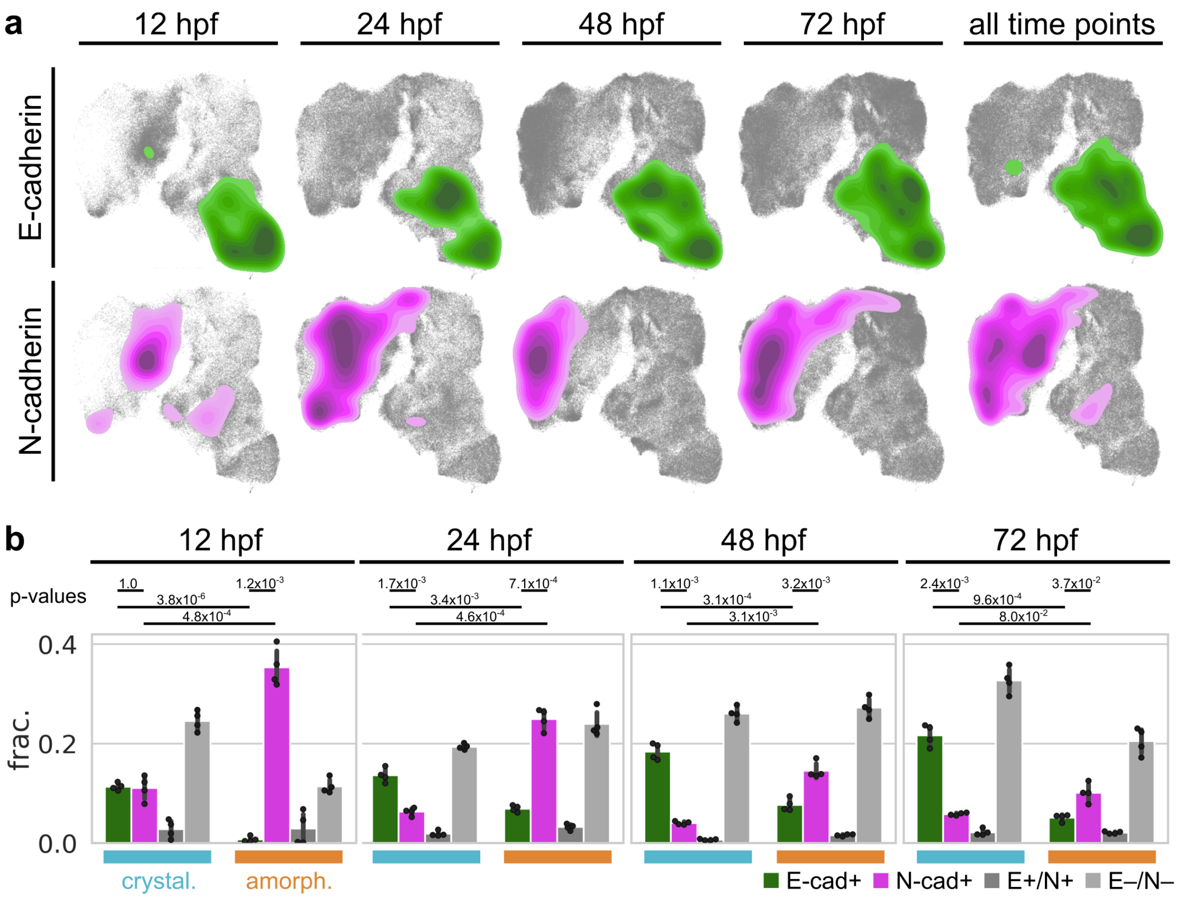

Supplementary Figure 17: E- and N-cadherin associate with crystalline and amorphous archetype during early development

a: E- and N-cadherin localise on opposing ends of organisational feature space at all analysed time points, indicating consistently different tissue organisation. **b**: E- and N-cadherin consistently associate with crystalline and amorphous archetypes. Fraction of E-/N- cadherin expressing cells that organise crystalline/amorphous is significantly higher than both E-/N-cadherin expressing cells that organise amorphous/crystalline, and N-/E-cadherin expressing cells that organise crystalline/amorphous. Total number of samples N=16 (4 per time point), total number of cells n=1,698,230. Statistical test: Welch's t-test independent samples with Bonferroni correction. Source data are provided as a Source Data file.

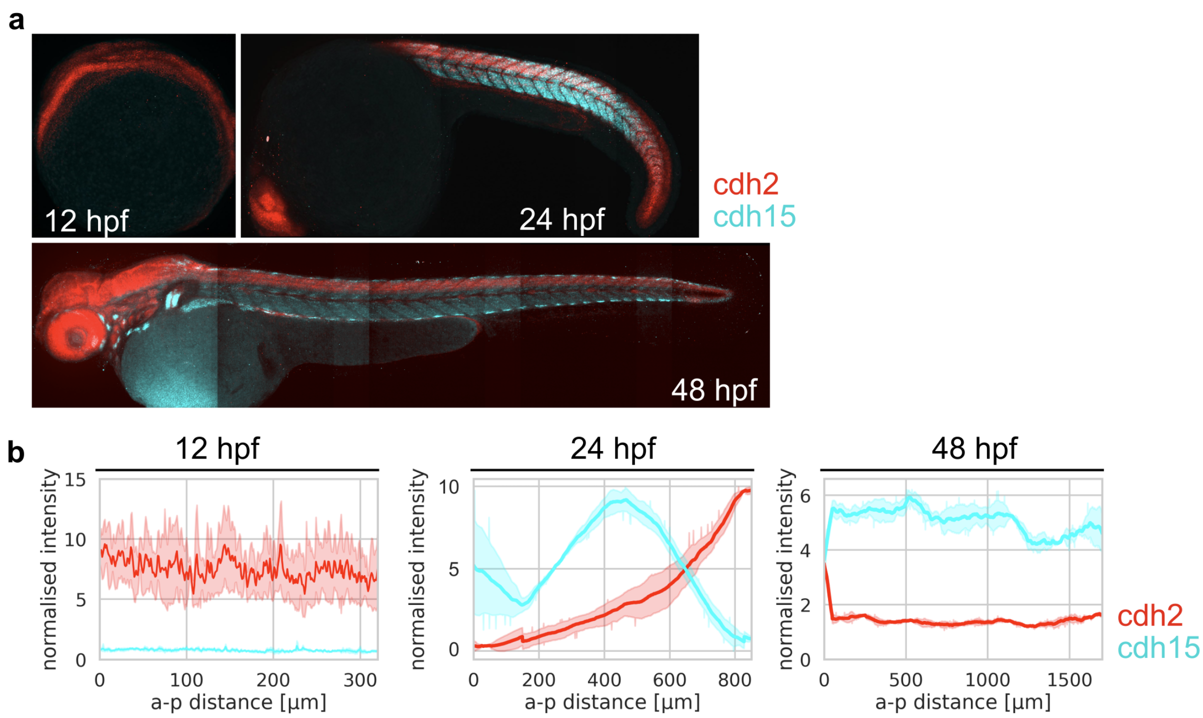

Supplementary Figure 18: During muscle development cdh15 and N-cadherin are expressed in an opposing fashion.

N-cadherin is expressed in developing muscles at 12 hpf, exhibits an anteroposterior gradient in the posterior region at 24 hpf and is fully absent at 48 hpf. Cdh15 shows an opposing pattern. **a**: N-cadherin (cdh2) and cdh15 labelled with HCR RNA FISH at different time points. Maximum intensity projection. **b**: Quantification of cadherin expression in the muscles along the anteroposterior axis. Intensity values are normalised to non-specific fluorescence in the sample (yolk). Error bands indicate 90% confidence interval, N=3 samples per time point. Source data are provided as a Source Data file.

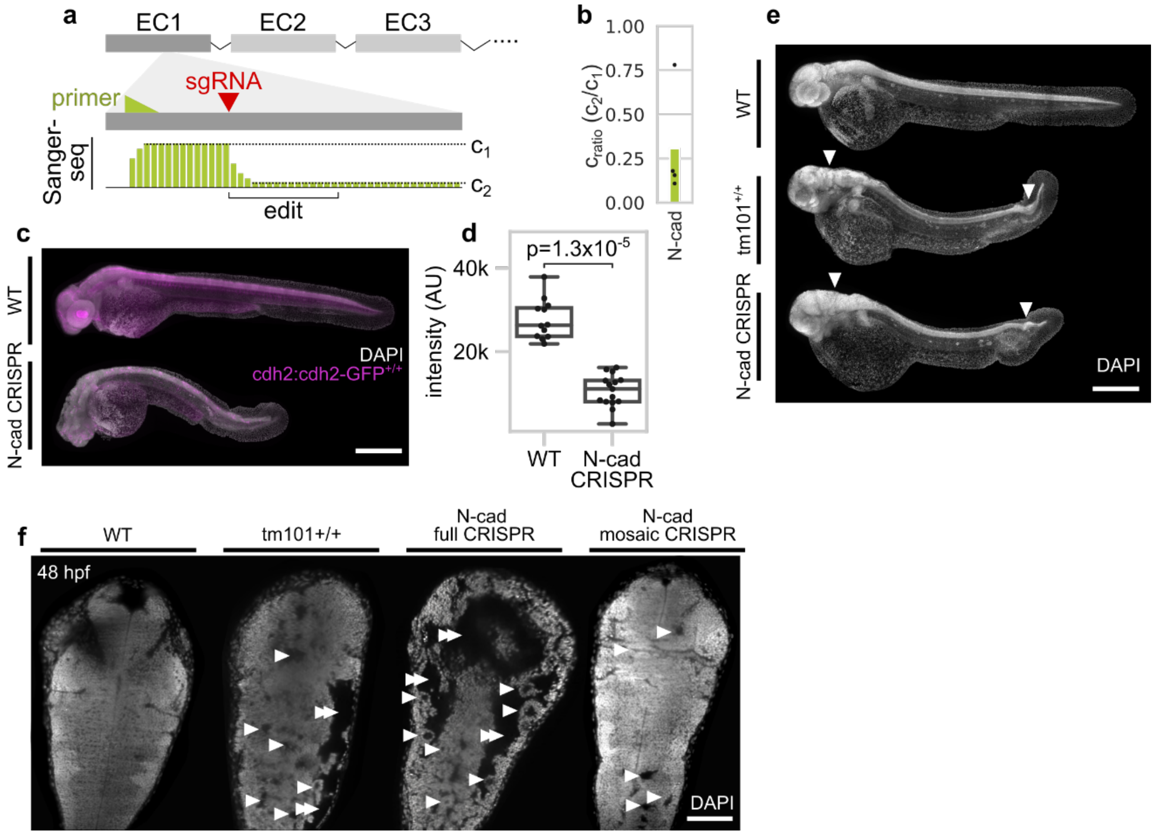

Supplementary Figure 19: CRISPR mediated, transient KO of N-cadherin is verified by sequencing and imaging, and phenocopies established stable mutant.

a: sgRNA for N-cadherin target regions within the first extracellular repeat (EC) domain. Edit region is amplified via PCR using a primer in proximity of the sgRNA target site and Sanger sequenced. Alignment to the target region exhibits drop in quality (c1 vs. c2) at the target site if edit was successful (**b**), rendering the protein non-sensical. **c, d**: Transient N-cadherin KO (N-cad CRISPR) leads to loss of N-cadherin fusion protein (BAC(cdh2:cdh2-GFP)) expression. Quantification based on n=12 WT samples and n=15 N-cad CRISPR samples. Statistical test: Mann-Whitney. **e**: N-cad CRISPR phenocopies the parachute mutant ($cdh2^{tm101}$). Arrowheads indicate regions exhibiting the strongest phenotype in the brain and tail. N=34 for WT, 12 for tm101+/+, 15 for N-cad CRISPR. **f**: KO of N-cad via CRISPR injection into 1-cell stage of embryonic development (full CRISPR) results in severe brain phenotype including loss of brain regions, cyst formation (arrowheads) and dramatic ventricular enlargement (double arrowhead). The parachute mutant (tm101) exhibits a similar, albeit slightly weaker phenotype. KO of N-cad via CRISPR injection at the 8-cell stage (mosaic CRISPR) largely omits this phenotype and leads to only minor defects in brain development compared to WT, tm101+/+ and full CRISPR (arrowheads). N=12 for WT, 12 for tm101+/+, 15 for N-cad full CRISPR, 15 for N-cad mosaic CRISPR. Scale bars: c, e: 400 μm, f: 100 μm. Source data are provided as a Source Data file.

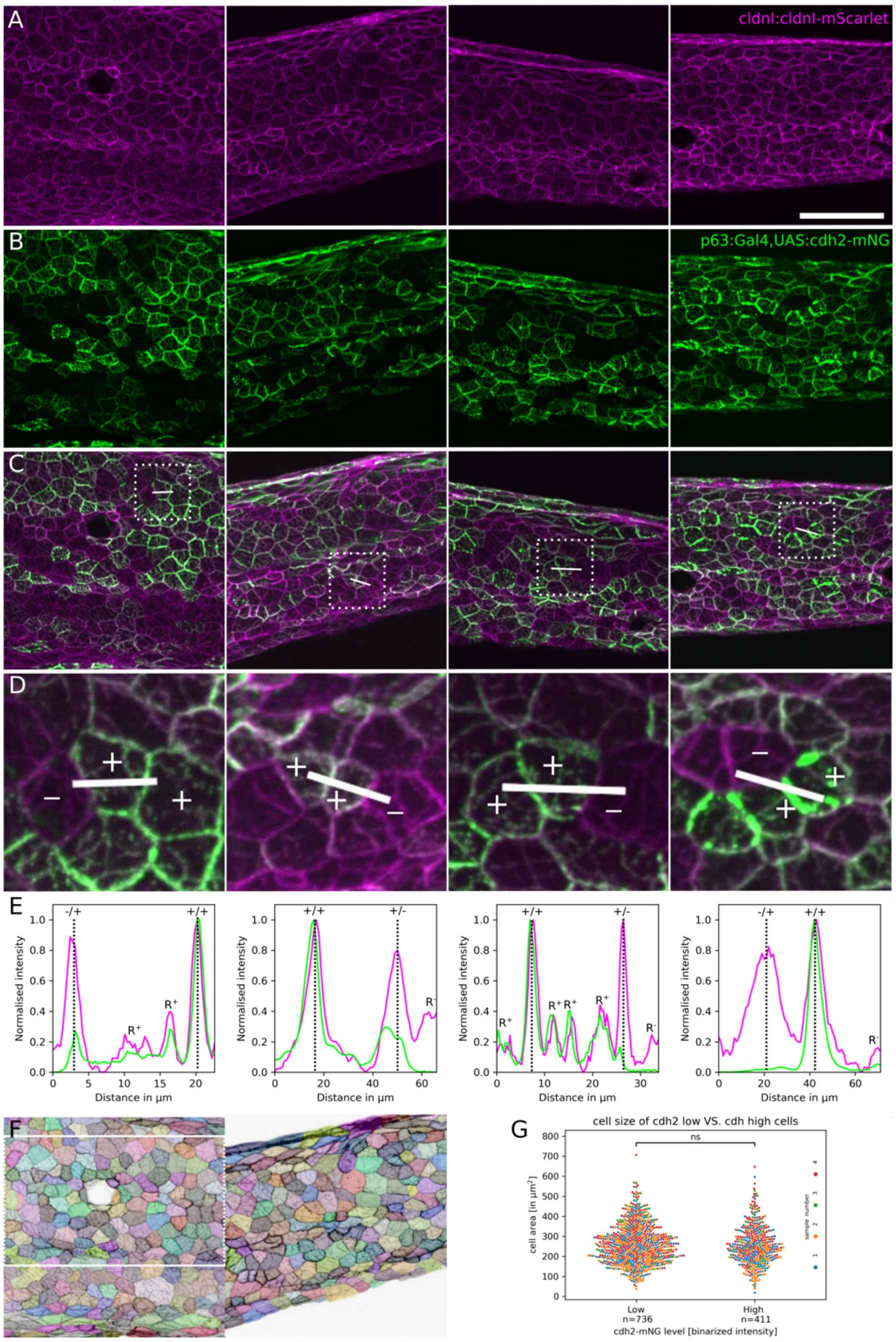

Supplementary Figure 20: N-cadherin overexpression in the skin does not change cellular morphology or organisation.

A: cldnl:cldnl-mScarlet (shown in magenta) marks the outlines of basal skin cells of 2dpf zebrafish larvae. Maximum projection, scale bar 100 µm. **B**: Overexpression of cdh2-mNG using p63:Gal4, UAS:cdh2-mNG Gal4-UAS in basal skin cells (green). Maximum projections. **C**: Overlay of both channels from A and B. The dotted regions show the location of the close-ups in C. **D**: Close-ups of overlay of both channels. Some cells are annotated as cdh2 high (+) or cdh2 negative/low (-). While cldnl is high at all cell outlines, cdh2 specifically localises to cell borders between positive cells. White lines indicate intensity line profiles in E. **E**: Intensities along the line profiles. Cell borders are labelled based on the cells' cdh2 levels as +/+ (cdh2 high-to-high), +/- (cdh2 high-to-low) or -/+ (cdh2 low-to-high). Ridges are labelled as R+ for ridges in cdh2 positive cells and R- for ridges in cdh2 negative cells. Intensities normalised to maximum and plotted in magenta and green for clndl:cldnl-mScarlet and p63:Gal4, UAS:cdh2-mNG. **F**: Segmentation of basal skin cells from cldnl:cldnl-mScarlet using cellpose2. Overlays of segmentation on top of grayscale cldnl images are shown. Box shows example of a region used for quantification. Dotted lines indicate direction where more fields of view were used for segmentation. **G**: Quantification of cell area split by cdh2 expression. No significant difference between cdh2 high and low expressing cells. Two-sided Mann-Whitney-Wilcoxon p-value=0.322, N=4. Source data are provided as a Source Data file.

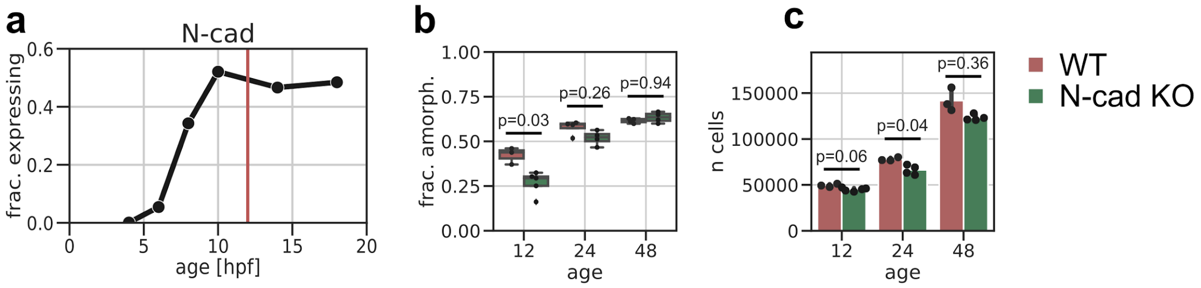

Supplementary Figure 21: Early N-cad expression and archetypal dynamics.

a: N-cadherin expression starts around 6 hpf of zebrafish development and rises sharply until 10 hpf, where it plateaus. Red line demarks 12 hpf, the first time point, where phenotypic analysis of N-cad CRISPR was conducted. Data from[14], n=29,608 cells. **b:** Fraction of amorphously organising cells between wildtype and CRISPR mediated N-cad knockout is lower at 12 hpf but nears parity at 48 hpf. **c:** Number of cells per sample is the same between WT and N-cad KO at 12 hpf. KO shows reduced cell count at 24 and 48 hpf, indicating gross developmental defects. Welch's t-Test. Sample number N=3 control, 4 mKO per time point in b, c. Source data are provided as a Source Data file.

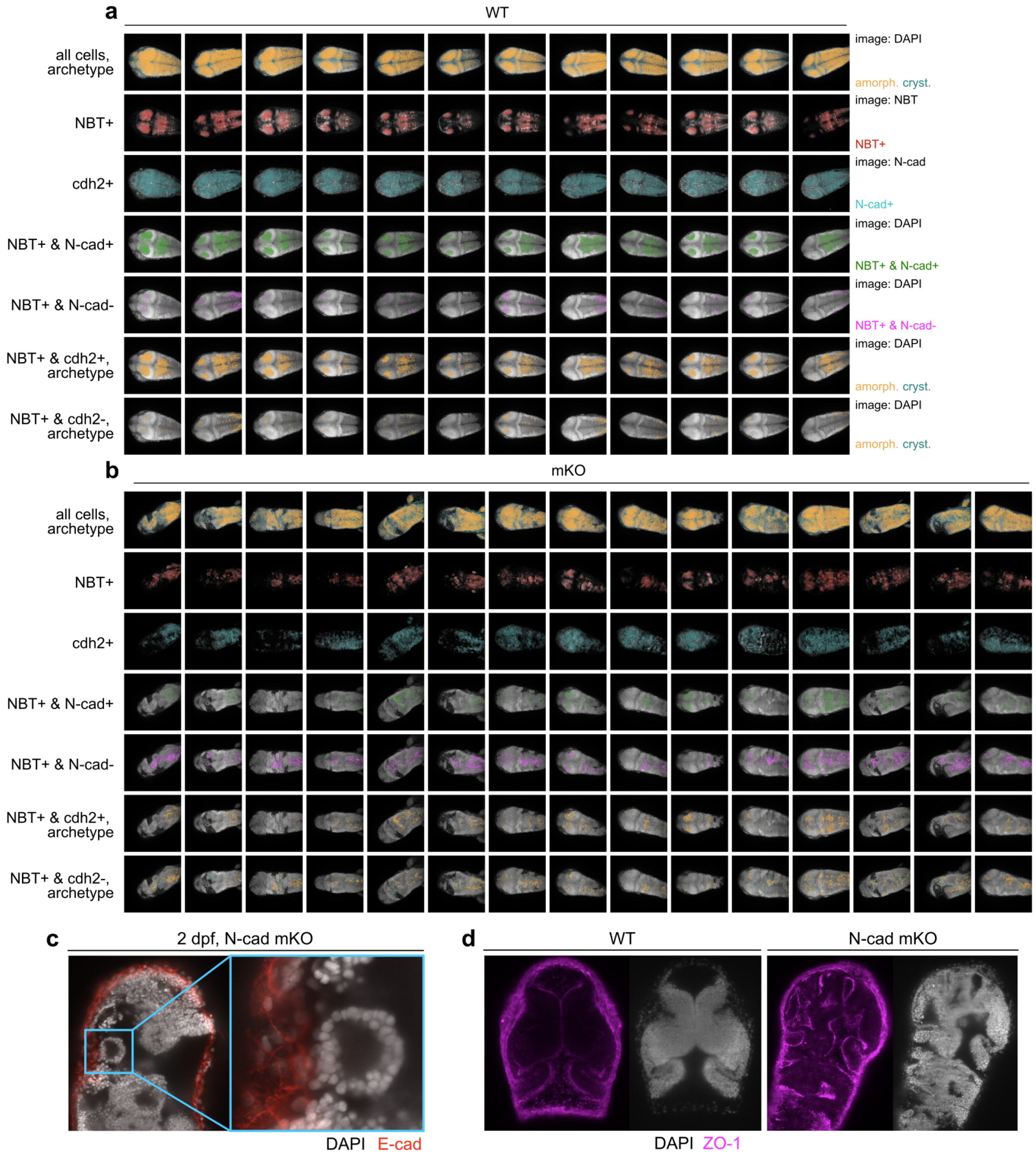

Supplementary Figure 22: Mosaic CRISPR of N-cadherin generates identifiable knockout and induces morphological phenotype

Maximum intensity projections of all imaging data used for the mosaic N-cadherin knockout experiments. Wildtype samples in **a** exhibit conventional tissue architecture in the brain, express NBT in a regular restricted pattern and express N-cadherin in a wider pattern. Classification of segmented nuclei based on gene expression works reliably. Co-expression domains of NBT and N-cadherin are regular, bilaterally symmetrical, and predominantly organised amorphously. Cells expressing only NBT are almost fully absent. mKO samples in **b** exhibit a visible morphological phenotype. Cells expressing only NBT are more frequent and organise more crystalline. **c**: N-cadherin deficient cells do not upregulate E-cadherin. Representative image of N=4 samples. **d**: N-cadherin mKO leads to broader expression of the epithelial marker ZO-1 in the 48 hpf zebrafish brain. Representative image of N=4 samples.

Supplementary Notes

Supplementary Note 1: Adaptively restricted Voronoi diagram

To generate features that captured the spatial organisation of the segmented nuclei, we needed to establish spatial neighbourhood relationships between them in a manner that reflected local cell arrangement. We used a modified version of the three-dimensional Voronoi diagram to achieve that. In a conventional Voronoi diagram, space is partitioned based on the distance between neighbouring points, by assigning all space that is closest to an individual point to its 'Voronoi cell', which consequently reflects local organisation in a robustly quantifiable way. This generates interfaces between Voronoi cells that are equidistant between two points, and vertices that are closest to three or more points. One challenge with the Voronoi approach is that it breaks down at external boundaries, since there Voronoi cells grow infinitely large (Suppl. Fig. 2 a,b).

A naïve approach to circumvent this would be to limit the size of Voronoi cells such that they cannot exceed a certain radius or volume. While this method is commonly used to remove giant Voronoi cells e.g., for visualisation, it is not suited for our purpose, since it biases boundary Voronoi cells to a pre-defined, maximal volume, making them incomparable to Voronoi cells from the inside. In fact, such cells differ significantly even from their close neighbours that are just one layer below (Suppl. Fig. 2 c).

Another approach to limit boundary Voronoi cells is to generate a surface mesh of the whole point cloud and to cut the boundary Voronoi cells with it[15–17]. Given a high-quality surface mesh, this method is very powerful, as it would conserve the surface shape of the sample. However, generating such a mesh from imaging data is difficult and would require specific genetic markers that may not be generally attainable. Deriving a surface mesh from the point cloud itself is also generally challenging. For point clouds with convex topology a surface mesh can be trivially generated from the convex hull. However, concave topologies cannot be isolated in such a rigorous way and would involve the use of parameterised methods such as alpha shapes that require tuning of 'concavity' parameters or similar[18]. Moreover, conventional meshing approaches are only sensitive to boundaries to the outside and would fail to detect cavities inside the point cloud[19].

To overcome these limitations, we developed a method in which individual Voronoi cells were adaptively size- and shape restricted using auxiliary seed points estimated from their neighbourhood. Initially, a naïve radially restricted Voronoi diagram was generated with restriction radius 50 µm, and boundary Voronoi cells were defined as cells with at least one face which was not shared with another Voronoi cell. For these boundary cells, all neighbouring cells were identified and their average number of neighbours n_n as well as their average volume V_n was determined. From this, n_n auxiliary points were placed isotopically, in the pattern of a Fibonacci lattice[20], on a sphere around every boundary nucleus at the distance $d_n = 2 \cdot \sqrt[3]{3V_n/(4\pi)}$. Subsequently, all auxiliary points that were placed outside the boundary Voronoi cell were deleted. This way auxiliary points were only placed in spots where they would restrict the Voronoi diagram towards the boundary. Finally, a second Voronoi diagram was generated on the combined set of nuclei and auxiliary points, resulting in restricted boundary cells (Suppl. Fig. 2 d). The auxiliary points were not considered for further processing.

To increase robustness, a k-nearest neighbour search was implemented in case a cell was surrounded by exclusively boundary cells. In that case, nearest neighbours would be iterated over starting with the closest and the n_n and V_n would be generated from the first five non-

boundary nearest neighbours. This method generated a Voronoi diagram in which boundary cells, and cells in sufficiently large cavities were restricted in a way that continued the distribution of the local neighbourhood. This enabled the comparison of inside and boundary cells in a common framework. Beyond the scope of this study, this method may generally be useful to construct restricted Voronoi diagrams on arbitrary point clouds without boundary mesh since it only requires the upper limit of a Voronoi cell's size as a single free parameter.

Supplementary Note 2: Definition of organisational features

Multi-scale cell density

A fundamental feature of tissue organisation is cell density. We measured cell density at different length scales to not only assess the compactness of a cell's immediate neighbourhood, but also the overall density of its surrounding tissue. To achieve this, we employed a kernel density estimation. For an individual cell, we centred on the coordinates of its nucleus and counted the number of segmented nuclei in a number of spheres with increasing radii (Suppl. Fig. 3 a). Then, we normalised the measured cell count by the analysed volume to make the values comparable between radii and convert the count into a density value. To identify an appropriate set of radii, we evaluated the correlation between measured densities at different radii. This indicated a strong correlation between consecutive spheres. Measuring cell density in stacked spherical shells rather than solid spheres decreased the correlation between measurements at different radii. Therefore, we chose this geometry as the basis of multi-scale density estimation. Moreover, we selected the radii 10 µm, 20 µm and 30 µm. These were selected since cells have a stereotypic diameter of ~10 µm, which led to a bias towards 0 μm^{-3} measurements in low-density areas for smaller radii, and spherical shells exceeding a radius of 30 µm exhibited correlation exceeding 60%, indicating mutual information content for larger radii (Suppl. Fig. 3 b).

Summarising, we estimated local cell density per segmented nucleus by counting the number of segmented nuclei within spherical shells between 0-10 µm (solid sphere), 10-20 µm, and 20-30 µm, normalising the count by the measured volume. This yielded a local, volumetric density estimation $[\,]_{msd}$ following the equation

$$[\,]_{msd} = \frac{3\, n_{points}}{4\,\pi(R^3 - r^3)} \quad (1)$$

with the number of counted points per volume points n_{points}, the inner sphere radius r and the outer sphere radius R. Evaluating cell density at different length scales was key to evaluate, for example, whether densely packed cells were a compact cluster in an otherwise sparse context, or if the dense packing was the overall tissue organisation.

Voronoi cell features

We used the restricted Voronoi diagram to quantify the local distribution of segmented nuclei. The Voronoi diagram is specifically appropriate for this, as it encodes the local distribution of points in the shape and size of individual Voronoi cells. Therefore, quantifying those parameters effectively characterises the local point distribution in a point-by-point way. Moreover, the Voronoi diagram generates a spatial hierarchy, i.e., relationships between points, in a mathematically rigorous way, making it both robust and generally applicable. The following organisational features were measured directly on Voronoi cells and were together used to characterise the local organisation of individual cells.

Voronoi cell volume

The volume of a Voronoi cell anti-correlates with the density of its neighbourhood, with the volume being lower in denser regions and higher in less dense regions. Moreover, it also measures the anisotropy of the local neighbourhood since it will be lower if density is high in only one direction. One way to illustrate this would be to imagine a Voronoi cell as an ellipsoid

whose volume $V_{ellipsoid}$ would be given by $V_{ellipsoid} = \frac{4}{3}\pi abc$ with a, b, c being the length of the three semi-axes. If any one of them gets close to 0, the whole volume will go to 0.
The volume of the Voronoi cells was calculated via a tetrahedral decomposition[21] of the Voronoi cell. The volume of the tetrahedra was subsequently determined via

$$V_{tetra} = \frac{|\tilde{a}\cdot(\tilde{b}\times\tilde{c})|}{6}; \qquad \tilde{a}, \tilde{b}, \tilde{c} = [a, b, c] - d \quad (2)$$

with a, b, c, d being the vectors pointing to each vertex of the tetrahedron. The sum of all tetrahedral volumes was the total Voronoi cell volume (Suppl. Fig. 3 c).

Voronoi density
To estimate local cell density in a non-kernel-based way, we measured the distance between individual nuclei and their adjacent nuclei, i.e., nuclei of Voronoi cells sharing an interface (Suppl. Fig. 3 c). Sharing an interface in the Voronoi diagram is equivalent to being connected on the Delaunay graph. The average inverse distance between cells in a Delaunay neighbourhood described local cell density, while the standard deviation of that distribution measured the regularity of density, with higher values indicating more irregularity.

Number of neighbours
Since the Voronoi diagram establishes neighbourhood relationships between unstructured points, the number of neighbours of any given point is a key feature. We measured the number of neighbours by counting the number of connections of a point (node) on the Delaunay graph, i.e., the number of shared faces per Voronoi cell (Suppl. Fig. 3 c). Note that faces to the outside, i.e., restricted faces, were not counted. Shared interfaces were not weighted by interface area and, therefore, it was more likely for an irregularly arranged neighbourhood to exhibit a higher number of neighbours.

Centroid offset
To measure the isotropy of local point arrangements, we measured the Euclidean distance between a Voronoi cell's seed point—the location of the nucleus—and its centroid. The location of the centroid was determined via tetrahedral decomposition of the Voronoi cell. In an isotropic environment, the seed point and centroid of a Voronoi cell are identically localised, while their distance increases as a function of local anisotropy (Suppl. Fig. 3 c).

Quantification of local organisational variability
To evaluate the variability of feature values in a local neighbourhood, we analysed the distribution of Voronoi cell volume, number of neighbours, and centroid offset in the Voronoi neighbourhood of each segmented nucleus. From that, we calculated mean and standard deviation of the distributions as additional feature values. Importantly, the segmented nucleus used to generate that neighbourhood was excluded from the analysis. This way we were able to contrast the feature value of an individual cell, e.g., its number of neighbours, with the average feature value of the neighbourhood. Moreover, we gained access to the overall feature variability in a cell's neighbourhood via the standard deviation (Suppl. Fig. 3 d). Overall, this neighbourhood analysis expanded our feature set by six neighbourhood features that mainly carry information about local heterogeneity.

Normalisation enables cross-feature comparison

To enable further analysis, such as low dimensional embedding and clustering, and to make features comparable between each other, individual feature values were normalised using the standard z-scaling approach:

$$\widetilde{f_i} = \frac{f_i - \langle f \rangle}{\sigma_f} \quad (3)$$

With the individual feature value f_i, the normalised feature value $\widetilde{f_i}$, the mean feature value across the samples $\langle f \rangle$ and the corresponding standard deviation σ_f. We found this scaling approach more robust than a scaling of the minimum and maximum feature value between 0 and 1 since it is less biased towards outliers. The feature normalisation was performed jointly on all samples to conserve sample-to-sample variability and differences between experimental conditions.

Supplementary Note 3: Gaussian Mixture Model stratifies organisational feature space into eleven motifs

To identify organisational motifs, we clustered cells based on the similarity of their organisational features. A Gaussian Mixture Model (GMM) was used to approximate the distribution of cells in the organisational feature space. In short, such a model uses a pre-defined number of n-dimensional Gaussian distributions and optimises their coordinates as well as their covariance matrices to best fit a given point distribution. In this context, points are treated as observations, while the GMM models the underlying probability density function. The parameters of the GMM are optimised to maximise the likelihood of the observations under the probability density function[22].

We elected to use a GMM for several reasons. Compared with simpler methods such as K-Means clustering, GMMs generally identifies clusters in data more reliably and is able to model finer-grained distributions more accurately[23]. Moreover, the probabilistic nature of the GMM enabled us to quantitatively assess the model performance on our data. More advanced clustering techniques such as DBSCAN[24] or OPTICS[25] are most sensitive to discrete clusters and hence were unable to identify meaningful clusters in our largely continuous data. Moreover, those methods were computationally significantly more expensive than the GMM.

The free parameter of a GMM is its number of components, i.e., how many Gaussian distributions will be used to approximate a given point distribution. Setting this number correctly is crucial for a good model fit. To determine the number of components, we optimised GMMs with number of components between 2 and 34 and measured the model performance at every iteration by analysing the model's Bayesian Information Criterion, its Silhouette Score and its Jensen-Shannon divergence.

The Bayesian Information Criterion BIC was defined as

$$BIC = kln(n) - 2ln\left(\hat{L}(x)\right) \quad (4)$$

with the maximised likelihood function $\hat{L}$, observations x, number of model parameters k and the number of observations n (Wit et al., 2012). Low BIC scores indicate a good model fit. However, reportedly, the penalty term of the BIC score (left) is insufficient to suppress a bias towards models with too many components. Therefore, we evaluated the gradient of the BIC between consecutive numbers of components.

The Silhouette Score was defined as the mean Silhouette Coefficient s_i across all points in each cluster.

$$s_i = \frac{b_i - a_i}{max\,(a_i, b_i)} \quad (5)$$

With the mean intra-cluster distance of the point a_i and the nearest-cluster distance b_i [26]. Consequently, the Silhouette Score is in the range [-1, 1] with values close to 1 indicating a good fit of the clustering model. Similarly to the BIC, the Silhouette Score often biases analysis towards complex models, which can be circumvented by analysing its gradient.

For the Jensen-Shannon divergence, the dataset was randomly split into two sets of equal size and an n-component GMM was generated on each of them individually. The probability distributions P, Q of the two GMMs were estimated by converse random sampling of 20,000 points, i.e. for set X drawn from P, the log probabilities were $log\,log\,(P(x))$ and $log\,log\,\left(Q(x)\right)$ with $x \in X$, and for set Y drawn from Q, the log probabilities were $log\,log\,(P(y))$ and $log\,log\,\left(Q(y)\right)$ with y$\in Y$. From this the Jensen-Shannon divergence was calculated as

$$JS = \frac{1}{2} \cdot \left((2) \ + \left\langle log \log\left(P(x)\right) \right\rangle_{x \in X} - \langle log \log(P(x) + Q(x) \rangle_{x \in X} + \left\langle log \log\left(Q(y)\right) \right\rangle_{y \in Y} - \langle log \ log \ (P(y) + Q(y) \ \rangle_{y \in Y} \right)$$

(6)

With the mean over a set $\langle \ \rangle_{x,y \in X,Y}$. A low JS indicates that the two GMMs of the split dataset are similar and hence the number of components fits well with the data[9].
To reliably estimate the number of components, the consensus of these three metrics over 100 iterations based on 80,000 randomly selected data points was used. From this analysis we determined the number of GMM components to be eleven (Suppl. Fig. 7).

Supplementary Tables

Table 1: Parameter values used for TGMM 2.0 nuclear segmentation

The parameters ‘imageFilePattern’ and ‘backgroundThreshold’ were set for each preprocessed image individually.

Parameter	Value
anisotropyZ	1
persistanceSegmentationTau	5
betaPercentageOfN_k	0.05
nuPercentageOfN_k	1
alphaPercentage	0.7
maxIterEM	100
tolLikelihood	1.00E-06
regularizePrecisionMatrixConstants_lambdaMin	0.02
regularizePrecisionMatrixConstants_lambdaMax	0.1
regularizePrecisionMatrixConstants_maxExcentricity	9
temporalWindowForLogicalRules	5
thrBackgroundDetectorHigh	1.1
thrBackgroundDetectorLow	0.2
SLD_lengthTMthr	5
radiusMedianFilter	1
minTau	2
conn3D	74
estimateOpticalFlow	0
maxDistPartitionNeigh	80
deathThrOpticalFlow	-1
minNucleiSize	8
maxNucleiSize	3000
maxPercentileTrimSV	0.2
conn3DsvTrim	6
maxNumKNNsupervoxel	10
maxDistKNNsupervoxel	41
thrSplitScore	-1
thrCellDivisionPlaneDistance	12.403
thrCellDivisionWithTemporalWindow	0.456

Table 2: Single guide RNA (sgRNA) sequences for cadherin deletion

Pairs of sgRNAs were designed in close proximity to each other to induce large sequence deletions. sgRNAs marked with * are published in[27]. Italicised sgRNAs were identified as non-functional. The number of base pairs between the sgRNAs Δ was not relevant for cdh2, since their distance was too large for efficient whole-sequence deletion (> 60 kbp).

gene	sgRNA 1	sgRNA 2	Δ [bp]
cdh1*	GGGATCAAATTACTACCAAC	AGGATTGCTCAAACAGAGGT	630
cdh11	GGTGTCTCGAGCCACAGCCT	GCGCATAGTAAGGGCCGTGA	94
cdh13	TAACTTGCCACTCAGGTCAG	GGAGCCACGTTACTCTGGGG	80
cdh15	AGGGTCATCAAAGTCCACCG	TAGGCTGGAATTTGGGAGCG	334
cdh17	ATTGTTCGGGCTGAGGATTT	CTCATTGAGCAGTACGACAC	38
cdh18a	GCGAGACGTGTTGATTCGTA	CTTGTCGCCGTTGGGCAGTT	312
cdh2*	TAAACGATGTACCGTTCCGG	GGGACTCCAGCCTGGAATGC	---
cdh26.2	CACTCAGGCGATGGACTACG	ACCGGCACGTTTTGACGGG	212
cdh4	GAATGGGGATGTGAGGCGTC	CGCAGCGGGCATCGACAGAG	80
cdh5	TAGTGGCAGAATTCCCAGTC	AATTGGCCTTTTAGAGGTGG	219
cdh6	ACGGTGATGTACGTCCTCAC	TTACCAATGTGAGACCCCTC	235
cdh7a	CGTGCTTCTCGGTCCATGTT	TTGGCCACCACCGACGCCAC	642

Table 3: Primers used to validate CRISPR gene deletions

gene	primer forward	primer reverse
cdh1	agtgtcacagggaaatgaaaaca	cctctttcagcaatggttcaga
cdh11	ttcaggggaaggagcaggta	catgaggaacaagctgtcagg
cdh13	tgctgccaattagtctttccg	gcactgagttccatcaaacatca
cdh15	tgtgtttgtgtgcgatcagg	ccaacctccgcaatcagttt
cdh17	tggcatgaacagaaagaagaaca	ctgtggcaagatacatagggt
cdh18a	tgtcctctttccttcacacaga	gcccaatgtgctcttccaaa
cdh2-1	cttcttcgctctgacttccg	ccttcaggcaaaagcattcg
cdh2-2	tgatcatccaggctaccgatat	ttaacaacggtgacaaggcc
cdh26.2	cagtgaccttcaaattgcagat	tgagattgaaagaaacaccaggt
cdh4	ggtaacggcctttgacgc	agggtgagtaaacgatgcct
cdh5	gaaaagtgacctcgaccgtg	tgaggcttagcattccatctt
cdh6	cctattgctgcattcccctg	ttcactggtcctcacaagac
cdh7a	catacatcattaacggtgcatat	ccttcctgggtatctttgtct

1. Hummel, R. Image enhancement by histogram transformation. *Comput. Graph. Image Process.* **6**, 184–195 (1977).
2. Lucy, L. B. An iterative technique for the rectification of observed distributions. *Astron. J.* **79**, 745 (1974).
3. Richardson, W. H. Bayesian-Based Iterative Method of Image Restoration*. *JOSA* **62**, 55–59 (1972).
4. Amat, F. *et al.* Efficient processing and analysis of large-scale light-sheet microscopy data. *Nat. Protoc.* **10**, 1679–1696 (2015).
5. McDole, K. *et al.* In Toto Imaging and Reconstruction of Post-Implantation Mouse Development at the Single-Cell Level. *Cell* **175**, 859-876.e33 (2018).
6. Scott, D. W. *Multivariate Density Estimation*. (John Wiley & Sons, Inc, 2015).
7. Ward, J. H. J. Hierarchical Grouping to Optimize an Objective Function. *J. Am. Stat. Assoc.* **58**, 236–244 (1963).
8. McLachlan, G. & Rathnayake, S. On the number of components in a Gaussian mixture model. *WIREs Data Min. Knowl. Discov.* **4**, 341–355.
9. Nielsen, F. On the Jensen–Shannon Symmetrization of Distances Relying on Abstract Means. *Entropy* **21**, 485 (2019).
10. Farnsworth, D. R., Saunders, L. M. & Miller, A. C. A single-cell transcriptome atlas for zebrafish development. *Dev. Biol.* **459**, 100–108 (2020).
11. Jumper, J. *et al.* Highly accurate protein structure prediction with AlphaFold. *Nature* **596**, 583–589 (2021).
12. Varadi, M. *et al.* AlphaFold Protein Structure Database: massively expanding the structural coverage of protein-sequence space with high-accuracy models. *Nucleic Acids Res.* **50**, D439–D444 (2021).
13. Kearse, M. *et al.* Geneious Basic: An integrated and extendable desktop software platform for the organization and analysis of sequence data. *Bioinformatics* **28**, 1647–1649 (2012).
14. Wagner, D. E. *et al.* Single-cell mapping of gene expression landscapes and lineage

in the zebrafish embryo. *Science* **360**, 981–987 (2018).

15. Sainlot, M., Nivoliers, V. & Attali, D. Restricting Voronoi diagrams to meshes using corner validation. *Comput. Graph. Forum* **36**, 81–91 (2017).

16. Wang, P. *et al.* Robustly computing restricted Voronoi diagrams (RVD) on thin-plate models. *Comput. Aided Geom. Des.* **79**, 101848 (2020).

17. Yan, D.-M., Wang, W., Lévy, B. & Liu, Y. Efficient Computation of 3D Clipped Voronoi Diagram. in *Advances in Geometric Modeling and Processing* (eds. Mourrain, B., Schaefer, S. & Xu, G.) 269–282 (Springer, Berlin, Heidelberg, 2010). doi:10.1007/978-3-642-13411-1_18.

18. Gardiner, J. D., Behnsen, J. & Brassey, C. A. Alpha shapes: determining 3D shape complexity across morphologically diverse structures. *BMC Evol. Biol.* **18**, 184 (2018).

19. Abdelkader, A. *et al.* VoroCrust: Voronoi Meshing Without Clipping. *ACM Trans Graph* **39**, 23:1-23:16 (2020).

20. González, Á. Measurement of Areas on a Sphere Using Fibonacci and Latitude–Longitude Lattices. *Math. Geosci.* **42**, 49–64 (2010).

21. Bukenberger, D. R. & Lensch, H. P. A. Tetrahedra of varying density and their applications. *Vis. Comput.* **37**, 2447–2460 (2021).

22. Reynolds, D. Gaussian Mixture Models. in *Encyclopedia of Biometrics* 659–663 (Springer, Boston, MA, 2009). doi:10.1007/978-0-387-73003-5_196.

23. Patel, E. & Kushwaha, D. S. Clustering Cloud Workloads: K-Means vs Gaussian Mixture Model. *Procedia Comput. Sci.* **171**, 158–167 (2020).

24. Ester, M., Kriegel, H.-P., Sander, J. & Xu, X. A density-based algorithm for discovering clusters in large spatial databases with noise. in *Proceedings of the Second International Conference on Knowledge Discovery and Data Mining* 226–231 (AAAI Press, Portland, Oregon, 1996).

25. Ankerst, M., Breunig, M. M., Kriegel, H.-P. & Sander, J. OPTICS: ordering points to identify the clustering structure. *SIGMOD Rec* **28**, 49–60 (1999).

26. Rousseeuw, P. J. Silhouettes: A graphical aid to the interpretation and validation of

cluster analysis. *J. Comput. Appl. Math.* **20**, 53–65 (1987).

27. Wu, R. S. *et al.* A Rapid Method for Directed Gene Knockout for Screening in G0 Zebrafish. *Dev. Cell* **46**, 112-125.e4 (2018).
