# Supplementary material for: In toto analysis of embryonic organisation reduces tissue diversity to two archetypes requiring specific cadherins
Source: Nat Commun. 2025 Jul 25;16:6872. doi: 10.1038/s41467-025-62127-9 (PMC12297461; doi:10.1038/s41467-025-62127-9)
Supplement: Supplementary file 2 — Description Of Additional Supplementary File [file 41467_2025_62127_MOESM2_ESM.pdf]

Description of Additional supplementary files

Suppl. Dataset 1:

Interactive 3D rendering of 12 hpf zebrafish embryo. Spheres show nuclear position and colours indicate organisational archetype (orange: amorphous, cyan: crystalline).

Suppl. Dataset 2:

Interactive 3D rendering of 24 hpf zebrafish embryo. Spheres show nuclear position and colours indicate organisational archetype (orange: amorphous, cyan: crystalline).

Suppl. Dataset 3:

Interactive 3D rendering of 48 hpf zebrafish embryo. Spheres show nuclear position and colours indicate organisational archetype (orange: amorphous, cyan: crystalline).

Suppl. Movie 1:

N-cadherin expression pattern at 12 hpf. Volumetric light sheet image of 12 hpf zebrafish embryo. White: DAPI, magenta: Ncadherin. Scale bar: 500 µm.

Suppl. Movie 2:

N-cadherin expression pattern at 24 hpf. Volumetric light sheet image of 24 hpf zebrafish embryo. White: DAPI, magenta: Ncadherin. Scale bar: 500 µm.

Suppl. Movie 3:

N-cadherin expression pattern at 48 hpf. Volumetric light sheet image of 48 hpf zebrafish embryo. White: DAPI, magenta: Ncadherin. Scale bar: 500 µm.
