# Supplementary material for: In toto analysis of embryonic organisation reduces tissue diversity to two archetypes requiring specific cadherins
Source: Nat Commun. 2025 Jul 25;16:6872. doi: 10.1038/s41467-025-62127-9 (PMC12297461; doi:10.1038/s41467-025-62127-9)
Supplement: Supplementary file 4 — Supplementary Data 2 [file 41467_2025_62127_MOESM4_ESM.html]

K3D snapshot viewer - Tue, 01 Apr 2025 18:14:41 GMT
