# Supplementary material for: In toto analysis of embryonic organisation reduces tissue diversity to two archetypes requiring specific cadherins
Source: Nat Commun. 2025 Jul 25;16:6872. doi: 10.1038/s41467-025-62127-9 (PMC12297461; doi:10.1038/s41467-025-62127-9)
Supplement: Supplementary file 9 — Reporting summary [file 41467_2025_62127_MOESM9_ESM.pdf]

nature portfolio

Corresponding author(s): Darren Gilmour, Max Brambach

Last updated by author(s): May 5, 2025

Reporting Summary

Nature Portfolio wishes to improve the reproducibility of the work that we publish. This form provides structure for consistency and transparency in reporting. For further information on Nature Portfolio policies, see our Editorial Policies and the Editorial Policy Checklist.

Statistics

For all statistical analyses, confirm that the following items are present in the figure legend, table legend, main text, or Methods section.

n/a	Confirmed	
☐	☒	The exact sample size (*n*) for each experimental group/condition, given as a discrete number and unit of measurement
☐	☒	A statement on whether measurements were taken from distinct samples or whether the same sample was measured repeatedly
☐	☒	The statistical test(s) used AND whether they are one- or two-sided<br>*Only common tests should be described solely by name; describe more complex techniques in the Methods section.*
☐	☒	A description of all covariates tested
☐	☒	A description of any assumptions or corrections, such as tests of normality and adjustment for multiple comparisons
☐	☒	A full description of the statistical parameters including central tendency (e.g. means) or other basic estimates (e.g. regression coefficient) AND variation (e.g. standard deviation) or associated estimates of uncertainty (e.g. confidence intervals)
☐	☒	For null hypothesis testing, the test statistic (e.g. *F*, *t*, *r*) with confidence intervals, effect sizes, degrees of freedom and *P* value noted<br>*Give P values as exact values whenever suitable.*
☒	☐	For Bayesian analysis, information on the choice of priors and Markov chain Monte Carlo settings
☒	☐	For hierarchical and complex designs, identification of the appropriate level for tests and full reporting of outcomes
☐	☒	Estimates of effect sizes (e.g. Cohen's *d*, Pearson's *r*), indicating how they were calculated

Our web collection on statistics for biologists contains articles on many of the points above.

Software and code

Policy information about availability of computer code

Data collection	Image acquisition was performed using Zen Blue (v2.1) for light sheet microscopy and Andor Solis for spinning disc confocal microscopy.
Data analysis	All analyses were performed using Python 3.7 with the following packages:<br>h5py (v3.2.1), k3d (v2.9.7), matplotlib (v3.5.3), numpy (v1.20.3), openTSNE (v0.5.1), pandas (v1.2.5), raster_geometry (v0.1.4.1), scikit-learn (v0.24.1), scipy (v1.7.3), seaborn (v0.11.1), setuptools (v58.0.4), tifffile (v2021.3.17), tqdm (v4.59.0), vedo (v2021.0.4), and vtk (v9.0.1).<br><br>Open source tools used include voro++ by Rycroft et al. for 3D Voronoi diagrams, MVRegFus by Albert et al. for multi-view fusion, Fiji (v2.90) for image analysis, TGMM2.0 from Philipp Keller's lab for nuclear segmentation, openTSNE (v1.0.2) for embedding, Scanpy (v1.9) for single-cell RNA-seq analysis, and AlphaFold (database version 2022-11-01) for protein structure prediction.<br><br>Commercial software: Geneious Prime (v2022.0) for sequence analysis.<br><br>All custom code used for analysis and figure generation is available publicly:<br>* nuQLOUD v1.0 [https://doi.org/10.5281/zenodo.15733475]<br>* TGMM_utility v1.0 [https://doi.org/10.5281/zenodo.15733494]

For manuscripts utilizing custom algorithms or software that are central to the research but not yet described in published literature, software must be made available to editors and reviewers. We strongly encourage code deposition in a community repository (e.g. GitHub). See the Nature Portfolio guidelines for submitting code & software for further information.

Data

Policy information about availability of data

All manuscripts must include a data availability statement. This statement should provide the following information, where applicable:

- Accession codes, unique identifiers, or web links for publicly available datasets
- A description of any restrictions on data availability
- For clinical datasets or third party data, please ensure that the statement adheres to our policy

All data supporting the findings of this study are available within this article, its supplementary files and the following online repositories.
The raw light sheet data (post multi-view fusion) generated in this study have been deposited in the BioImage Archive, accession number S-BIAD1405 [https://www.ebi.ac.uk/biostudies/bioimages/studies/S-BIAD1405?query=S-BIAD1405]65.
The scRNAseq data by Farnsworth et al. used in this study are available at NCBI SRA under accession code PRJNA564810 [https://www.ncbi.nlm.nih.gov/bioproject/564810].
The scRNAseq data by Wagner et al. used in this study are available at NCBI GEO under accession number GSE112294 [https://www.ncbi.nlm.nih.gov/geo/query/acc.cgi?acc=GSE112294].
Source data are provided with this paper.

Research involving human participants, their data, or biological material

Policy information about studies with human participants or human data. See also policy information about sex, gender (identity/presentation), and sexual orientation and race, ethnicity and racism.

Reporting on sex and gender	---
Reporting on race, ethnicity, or other socially relevant groupings	---
Population characteristics	---
Recruitment	---
Ethics oversight	---

Note that full information on the approval of the study protocol must also be provided in the manuscript.

Field-specific reporting

Please select the one below that is the best fit for your research. If you are not sure, read the appropriate sections before making your selection.

☒ Life sciences ☐ Behavioural & social sciences ☐ Ecological, evolutionary & environmental sciences

For a reference copy of the document with all sections, see nature.com/documents/nr-reporting-summary-flat.pdf

Life sciences study design

All studies must disclose on these points even when the disclosure is negative.

Sample size	Sample sizes of 3–4 biological replicates per condition were selected based on standard practice in the field for quantitative imaging of embryonic tissues. Although no formal power calculation was performed, sample sizes were sufficient to consistently observe the reported effects across independently acquired datasets. Reproducibility was ensured by repeating key experiments and confirming consistent trends across replicates.
Data exclusions	Data were excluded only in cases where image quality was insufficient to permit reliable segmentation. Exclusion decisions were based on manual inspection of the raw microscopy data prior to analysis, and were independent of experimental condition.
Replication	All key findings were independently replicated at least once, and all data from these experiments are included in the manuscript and supplementary materials.
Randomization	Data was not randomised.
Blinding	Blinding was not performed, as this is not common practice in the field for imaging-based developmental studies. Data analysis relied on automated and quantitative pipelines applied uniformly across all samples, which mitigates potential bias. Key findings were independently replicated using separate biological samples to ensure robustness.

Reporting for specific materials, systems and methods

We require information from authors about some types of materials, experimental systems and methods used in many studies. Here, indicate whether each material, system or method listed is relevant to your study. If you are not sure if a list item applies to your research, read the appropriate section before selecting a response.

Materials & experimental systems

n/a	Involved in the study
☒	☐ Antibodies
☒	☐ Eukaryotic cell lines
☒	☐ Palaeontology and archaeology
☐	☒ Animals and other organisms
☒	☐ Clinical data
☒	☐ Dual use research of concern
☒	☐ Plants

Methods

n/a	Involved in the study
☒	☐ ChIP-seq
☒	☐ Flow cytometry
☒	☐ MRI-based neuroimaging

Animals and other research organisms

Policy information about studies involving animals; ARRIVE guidelines recommended for reporting animal research, and Sex and Gender in Research

Laboratory animals	Danio Rerio (Zebrafish) Golden embryos age 12 to 72 hours post fertilisation.<br>Trensgenic zebrafish:<br>* Tg(NBT:dsRED), https://zfin.org/ZDB-FISH-150901-3075#summary<br>* TgCRISPR(cdh1-mLanYFP), https://zfin.org/ZDB-TGCONSTRCT-190508-6<br>* TgCRISPR(cdh1-tdTomato), https://zfin.org/ZDB-ALT-190419-2#summary<br>* TgBAC(cdh2:cdh2-GFP), https://zfin.org/ZDB-FISH-150901-25440<br>* TgBAC(p63:Gal4), https://zfin.org/ZDB-TGCONSTRCT-150424-2#summary<br>* TgCRISPR(cldni:cldni-mScarlett) x Tg(UAS:cdh2-mNG), this study<br>* cdh2(tm101/+), https://zfin.org/ZDB-FISH-150901-24840
Wild animals	No wild animals were used.
Reporting on sex	No sex determination was possible due to the age of the used embryos.
Field-collected samples	No field-collected samples were used.
Ethics oversight	All experiments were conducted in accordance with the regulation and guidelines of the veterinary office of the University of Zürich and the Canton of Zürich, Switzerland.

Note that full information on the approval of the study protocol must also be provided in the manuscript.

Plants

Seed stocks	---
Novel plant genotypes	---
Authentication	---
